# Effects of Inorganics during Hydrothermal Liquefaction of Waste: A Comprehensive Study

**DOI:** 10.1021/acs.energyfuels.5c05808

**Published:** 2026-02-09

**Authors:** Edoardo Tito, Marco Vitale, Giuseppe Pipitone, Samir Bensaid, Raffaele Pirone

**Affiliations:** Department of Applied Science and Technology, 19032 Politecnico di Torino, Corso Duca degli Abruzzi 24, 10129 Turin, Italy

## Abstract

Hydrothermal liquefaction (HTL) is gaining interest for the energy valorization of wet waste. While HTL performance is known to depend on biochemical composition, the role of inorganics remains poorly understood. This study evaluates the effects of the four most common metals (Na, K, Mg, and Ca) present as oxides, carbonates, phosphates, sulfates, and chlorides. Experimental results, supported by principal component analysis (PCA), revealed that inorganics significantly influence HTL performance, depending on both cation and anion type. More basic anions generally decreased solid production while favoring both biocrude and aqueous-phase yields, with carbonates performing better than oxides despite their lower basicity. Na and K enhanced these effects compared to Ca and Mg, while K and Ca led to higher HHVs and lower oxygen content in the biocrude than Na and Mg, respectively, indicating a specific role of the cations. Sodium and potassium carbonates performed best, increasing biocrude yield by 48% relative to the corresponding inorganic-free feedstock, while reducing solid production by 90%. CaCl_2_ was the only compound reducing biocrude yield, while increasing solid residue by 90%. This study highlights the critical influence of inorganics on HTL performance and provides a foundation for deeper insights into the underlying mechanisms.

## Graphical abstract

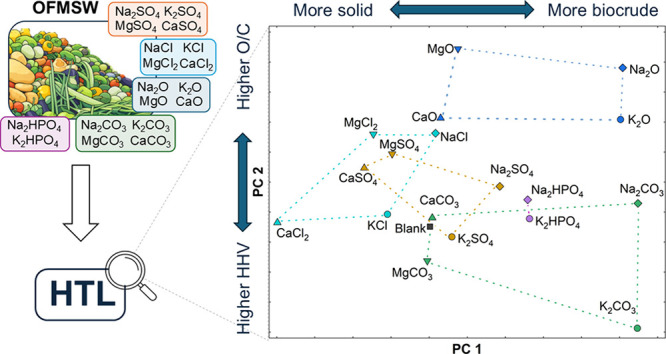

## 1 Introduction

It is estimated that around 2.1 billion tonnes of municipal solid waste were generated in 2020, with projections suggesting an increase to 3.8 billion tonnes by 2050.^1^ Over 50% of this waste is organic, primarily originating from green waste and food production and consumption.^1^ Effective management of municipal solid waste is critical, as it poses significant energy, economic, and social burdens worldwide, particularly in developing countries. Moreover, the urgent need to reduce greenhouse gas emissions highlights the importance of utilizing this waste to both minimize the volume requiring disposal and help meet the rising global energy demand. The production of biofuels, which is crucial for the decarbonization of heavy-duty transport, offers a potentially sustainable and impactful route for managing these wastes.

Within this context, hydrothermal liquefaction (HTL) is one of the promising processes for the production of an organic phase, called biocrude, which can subsequently be upgraded into biofuels.^2^ HTL mimics the natural biogeochemical processes responsible for petroleum formation but accelerates them to achieve biocrude production in less than 60 min. The process typically operates with dry solid loadings of 5–30 wt %, temperatures between 250 and 400 °C, and pressures ranging from 4 to 25 MPa.^3,4^ Under these conditions, the decreased dielectric constant and increased ionic product of water enhance biomass solubilization and facilitate the catalysis of ionic reactions, thereby improving process efficiency.^5^ Moreover, the water required for HTL aligns with the typical moisture content of organic waste feedstocks (∼70–85 wt %),^6^ making it energetically more favorable than dry thermochemical technologies such as gasification and pyrolysis.

A critical factor widely recognized in HTL is that process performance is strongly influenced by the type of feedstock used, its composition, and its characteristics.^7,8^ Indeed, numerous studies in the literature have examined the relationship between the biochemical composition of feedstocks and HTL performance.^9–11^ For instance, it is well established that biocrude yield generally follows the trend: lipids > proteins > carbohydrates. However, relatively little attention has been paid to the potential impact of the inorganic components contained therein, even though their effect may be non-negligible.^12,13^ Most studies addressing inorganics have focused on the intentional addition of specific metals to the feedstock to catalyze the HTL reaction, rather than investigating the influence of metals inherently present in the biomass. To this end, heterogeneous noble metals (Pd, Pt, Ru) and less expensive metals (Co, Mo, Ni), as well as other materials (zeolites, fly ashes, red mud, red clay) have been tested.^14–16^ Similarly, many investigations have focused on the use of basic homogeneous catalysts, primarily hydroxides and carbonates of alkali metals, which have generally enhanced biocrude production.^16–22^ In contrast, oxides, chlorides, and phosphates have received comparatively less attention.^16,20–25^ These studies typically employed a 1:20 catalyst-to-biomass mass ratio and tested a narrow selection of inorganic compounds. A notable exception is the work by Motavaf et al.,^16^ who explored a wide range of homogeneous and heterogeneous additives at a 1:2 catalyst-to-biomass mass ratio. Additionally, each paper focused on a different type of feedstock (micro- and macroalgae, manure, sawdust, straw, and food waste) making comparisons between the few inorganics tested in each of these works impossible. Food waste and the organic fraction of municipal solid waste (OFMSW) typically contain ash levels ranging from 2 to 15 wt %,^6^ with sodium (Na), potassium (K), magnesium (Mg), and calcium (Ca) being the most abundant inorganic metals.^26,27^ As these metals are inherently present in such wastes, it is essential to understand their impact on HTL performance.

To address this gap, the present study systematically evaluates the effects of these four common metal cations (Na, K, Mg, and Ca) in five typical chemical forms (oxides, carbonates, phosphates, sulfates, and chlorides), resulting in a total of 18 distinct inorganic species. All experiments were conducted at a fixed cation loading of 1 mol kg^–1^ of dry biomass, using a custom-made synthetic feedstock composed of cellulose, hen albumin, and sunflower oil, blended to simulate the biochemical composition of organic waste while minimizing background inorganic content. This controlled setup enables a clearer assessment of the direct catalytic effects of the cations and the influence of their associated chemical forms on HTL outcomes.

## 2 Experimental Section

### 2.1 Materials

Experiments were performed using cellulose (microcrystalline powder, Sigma-Aldrich), albumin from hen egg white (crude powder, Fluka), and sunflower oil (local supermarket) as representatives of carbohydrates, proteins, and lipids, respectively. Distilled water was used to prepare the starting solutions, while cyclohexane (Puriss. p.a., ACS reagent ≥ 99.5%, Sigma-Aldrich) and acetone (>99.5% GC, 32201-M, Sigma-Aldrich) were used as postreaction solvents.

The starting solutions were prepared with different inorganics purchased from Sigma-Aldrich, unless stated differently. These were sodium oxide (97%), potassium hydroxide (p.a., EMSURE, Supelco) calcium oxide (*ReagentPlus*, 99.9% trace metals basis), magnesium oxide (−10-+50 mesh, 98%), sodium carbonate (anhydrous, ACS reagent ≥ 99.5%), potassium carbonate (BioXtra, ≥ 99.0%), sodium chloride (BioXtra, ≥ 99.5% (AT)), potassium chloride (anhydrous, ACS reagent, ≥ 99%), calcium chloride (anhydrous granules Reag. Ph Eur), magnesium chloride (anhydrous for synthesis), sodium phosphate dibasic (puriss., meets analytical specification of Ph. Eur., BP, USP, FCC, E 339, anhydrous, 98–100.5% (calc. to the dried substance)), potassium phosphate dibasic (ACS reagent, ≥ 98%), sodium sulfate (puriss., meets analytical specification of Ph. Eur., BP, USP, anhydrous, 99.0–100.5% (calc. to the dried substance)), potassium sulfate (puriss. p.a., Fluka Chemical), magnesium sulfate (anhydrous, *ReagentPlus*, ≥ 99.5%).

Calcium sulfate was produced from a solution of calcium nitrate tetrahydrate (≥99.0%) via precipitation with sulfuric acid. The precipitated solid was washed with Milli-Q water, dried in an oven at 105 °C overnight, and calcined. Calcium carbonate was produced via carbonation of calcium oxide. A 1.2 L solution of 0.01 M CaO was flushed with 10% CO_2_ in He, at a flow rate of 100 mL/min under ambient temperature and pressure for 40 min. Magnesium carbonate (meets USP testing specifications) was dehydrated by calcination at 200 °C for 4 h and then at 220 °C for 1 h before use.

### 2.2 Reaction Step

Experiments were performed in 20 mL bomb-type reactors constructed from a 3/4″ 316 stainless steel tube, with both ends sealed by caps, as described in previous works.^28^ Reactors were loaded with 1.80 g dry biomass (1.78 g dry-ash free), 1.8 mmol cation-equivalent of inorganic additives, and distilled water to obtain a total slurry mass of 9 g. The starting composition of the biomass was: 63% cellulose, 19% albumin, and 18% sunflower oil, resembling the average composition of carbohydrates, proteins, and lipids in food waste and OFMSW, as determined from a preliminary literature survey reported in Figure S1. Distilled water and each inorganic additive were prepared and mixed prior to loading the reactor, obtaining a solution or suspension according to the solubility of the inorganic additive. The resulting solution/suspension was flushed with nitrogen for at least 1 h to remove absorbed CO_2_, without resulting in any reduction in the total volume. The list of the inorganic additives tested is reported in Table 1, along with their concentration in the flushed liquid solution/suspension, their theoretical pH, and the equivalent concentration in the biomass as ash and metal cations, as if they were already present. It is worth specifying that oxides readily convert into their hydroxide form as soon as they are dissolved in water.

**Table 1:** Inorganics Tested and Their Theoretical pH Values in Water at the Concentrations Used in This Work

		Liquid solution/suspension			Biomass	
Inorganic		Inorganic concentration (g/g)	pH	State at 25 °C	Ash equivalent (g/g_db_)	Metal equivalent (mg/kg_db_)
Chlorides	NaCl	1.46%	7.0	solution	5.52%	21 700
	KCl	1.86%	7.0	solution	6.94%	36 400
	MgCl_2_	2.38%	7.0	solution	8.69%	22 200
	CaCl_2_	2.77%	7.0	solution	9.99%	36 100
Sulfates	Na_2_SO_4_	1.78%	7.5	solution	6.63%	21 500
	K_2_SO_4_	2.18%	7.5	solution	8.01%	36 000
	MgSO_4_	3.01%	7.8	solution	10.74%	21 700
	CaSO_4_	3.40%	7.2	suspension	11.98%	35 300
Dibasic phosphates	Na_2_HPO_4_	1.77%	10.2	solution	6.63%	21 500
	K_2_HPO_4_	2.18%	10.2	solution	8.01%	36 000
Carbonates	Na_2_CO_3_	1.32%	11.7	solution	5.03%	21 800
	K_2_CO_3_	1.73%	11.7	solution	6.46%	36 600
	MgCO_3_	2.11%	10.7	suspension	7.78%	22 400
	CaCO_3_	2.50%	10.0	suspension	9.10%	36 400
Oxides	Na_2_O	0.77%	13.4	solution	3.01%	22 300
	K_2_O	1.18%	13.4	solution	4.50%	37 300
	MgO	1.01%	10.3	suspension	3.87%	23 400
	CaO	1.40%	12.7	suspension	5.31%	37 900

After loading, the reactors were submerged in a preheated sand bath (Techne SBL-2D, controller Techne TC-9D) at 350 °C. Within 160 s, the temperature inside the reactor reached 334 °C, representing 95% of the temperature change from 25 to 350 °C,^9^ which corresponds to a heating rate of approximately 115 K/min (Figure S2). Once the reactor reached 334 °C (red dot in Figure S2), it was held for a 30 min residence time before being removed from the sand bath and submerged in water for 5 min. The internal temperature dropped below 50 °C in less than 50 s.

### 2.3 Postreaction Workup

After cooling, the reactors were dried with compressed air, weighed, opened to vent the produced gas, and weighed again. The difference between these two measurements was used to determine the mass of gas produced. The reactor contents were then transferred into a 50 mL Falcon tube, and 5 mL of cyclohexane was added to the empty reactor. The reactor was sealed and manually shaken to dissolve any residual oil phase. The resulting cyclohexane solution was then transferred into the Falcon tube, which was manually shaken to ensure thorough contact with the solid phase. Next, the Falcon tube was centrifuged at 5000 rpm for 10 min, separating the contents into three distinct phases: a precipitated solid, an aqueous phase (AP), and a cyclohexane supernatant. Using Pasteur pipettes, the cyclohexane phase was transferred to a beaker, while the aqueous phase was collected in a 10 mL Falcon tube and stored in a refrigerator for later analysis. Subsequently, 5 mL of acetone was added to the empty reactor, which was then sealed, sonicated, and poured into the 50 mL Falcon tube containing the solid phase. The tube was sonicated again to facilitate interaction between the two phases, and the acetone solution was transferred to the beaker containing the cyclohexane. This process was repeated with additional 5 mL acetone aliquots until the solution became clear. At this stage, the contents of the beaker were vacuum-filtered. Any solid residue in the Falcon tube was transferred to the filter using a spatula and fresh acetone. The retained solid was then dried overnight in an oven at 105 °C, weighed, and collected. The combined apolar phase (cyclohexane and acetone) was dried with sodium sulfate and rotary-evaporated in a 50 mL round-bottom flask. Finally, the solid and biocrude samples were collected, weighed, and stored for further analysis.

### 2.4 Analysis

The mass yields of the different phases were calculated on a dry ash-free (daf) basis according to Eq. 1. The amount of ashes present in the feedstock and in the solids was evaluated as the residue obtained via thermogravimetric analysis (TGA, Mettler Toledo SDTA851) after the following temperature program: 25 °C (0 min hold) // 10 °C/min // 550 °C (30 min hold) under an air flow of 50 mL/min. For experiments with Ca-containing inorganics, the amount of CaCO_3_ present was evaluated by adding an additional ramp to the temperature program (20 °C/min from 550 to 900 °C under an airflow of 50 mL/min). The CaCO_3_ content was then calculated from the mass loss between 550 and 900 °C, as described in Eq. 2.

An elemental analyzer (Elementar Vario Macro Cube) was used to determine the elemental composition (CHNS) of the feedstock, biocrude, aqueous, and solid phases; oxygen was determined by difference from the measured C, H, N, S, and ash contents. For the solids, the carbon content was adjusted to remove the inorganic contribution arising from the decomposition of CaCO_3_ during the CHNS analysis (Eq. 3). The ash yield in the final solid was calculated according to Eq. 4, considering both the native ash content of the feedstock and the contribution from added inorganics. Higher heating values (HHVs) for the solid and biocrude phases were evaluated from the elemental analysis, following the method from Channiwala and Parikh.^29^ The carbon and nitrogen yield of the phases was calculated by dividing the masses of carbon and nitrogen in each phase by the corresponding content in the feedstock (Eq. 5 and Eq. 6). Energy recovery (ER) was calculated according to Eq. 7, with HHV values expressed in MJ/kg.

The compositions of the biocrude and aqueous samples were analyzed using a GC (Agilent 7890A GC) coupled with MS (Agilent 5975C). Biocrude samples were diluted 1:100 v/v in acetone, and 1 μL of the diluted sample was injected into a DB-5 ms column (30 m × 0.25 mm × 0.25 μm) in split mode with a split ratio of 20:1 and an injection temperature of 280 °C. The helium carrier gas flow rate in the column was maintained at 0.8 mL/min. The oven temperature program was as follows: 40 °C (5 min hold) // 10 °C/min // 100 °C (0 min hold) // 4 °C/min // 280 °C (0 min hold) // 10 °C/min // 300 °C (0 min hold). For the aqueous phase, 1 μL of syringe-filtered sample was injected into a DB-WAX Ultra Inert column (30 m × 0.25 mm × 0.25 μm) in split mode with a split ratio of 50:1 and an injection temperature of 240 °C. The helium carrier gas flow rate was maintained at 1 mL/min. The oven temperature program was as follows: 80 °C (1 min hold) // 5 °C/min // 250 °C (0 min hold). In both cases (biocrude and aqueous samples), compounds were identified using Agilent MassHunter Unknown Analysis software with the NIST 17 library and only compounds with a match factor greater than 70 and a peak area exceeding 1000 were considered. Identified compounds were classified into family groups based on a priority system, applied when a molecule could belong to more than one class. For biocrudes, the priority order was: long fatty acids > long fatty amides > long fatty nitriles > N-containing aromatics > aromatics > N-containing aliphatics > small fatty acids (C < 6) > cyclic ketones > other oxygenates (including alcohols, esters, linear ketones, and ethers) > aliphatic hydrocarbons. For the AP, the priority order was: pyridines > other N-containing aromatics > aromatics > cyclic imides > lactams > linear amides > other N-containing aliphatics > lactones > cyclic ketones > anhydrides > carboxylic acids > glycerol > other oxygenates (including alcohols, aldehydes, carbonates, esters, ethers, ketones, and oximes).

A preliminary boiling point distribution of the biocrudes was obtained via thermogravimetric analysis (TGA, Mettler Toledo TGA/DSC 3+) using the following temperature program: 25 °C (0 min hold) // 10 °C/min // 900 °C under an argon flow of 50 mL/min, followed by 5 min hold at 900 °C under an air flow of 50 mL/min.

Attenuated total reflectance Fourier-transformed infrared spectroscopy (ATR-FTIR) was performed using a Bruker Tensor 27 spectrometer. Twenty-four spectra in the range of 4000–400 cm^–1^ were automatically collected for each biocrude sample at a resolution of 2 cm^–1^, and the averaged spectrum was subsequently postprocessed using the baseline correction tool in OPUS software.

1 $${\mathrm{mass yield}}_{\mathrm{product},\mathrm{daf}}\left(\%\right)=\frac{{\mathrm{mass}}_{\mathrm{product},\mathrm{dry}}\left(1-\%{\mathrm{ash}}_{\mathrm{product}}\right)}{{\mathrm{mass}}_{\mathrm{feedstock},\mathrm{dry}}\left(1-\%{\mathrm{ash}}_{\mathrm{feedstock}}\right)}$$

2 $$\mathrm{CaC}O_{3\mathrm{solid}}\left(\%\right)=\frac{{\mathrm{mass}}_{\mathrm{loss between}\;550{}^{\circ}C-900{}^{\circ}C\;\mathrm{during TGA}}\left(g\right)}{{\mathrm{mass}}_{\mathrm{sample in TGA}}\left(g\right)}\times \frac{100.1}{44.0}$$

3 $${\mathrm{carbon}}_{\mathrm{solid}}\left(\%\right)={\mathrm{carbon}}_{\mathrm{from CHNS}}\left(\%\right)-\mathrm{CaC}O_{3\mathrm{solid}}\left(\%\right)\times \frac{12.0}{100.1}$$

4 $${\mathrm{ash yield}}_{\mathrm{solid}}\left(\%\right)={\mathrm{mass yield}}_{\mathrm{solid},\mathrm{dry}}\times \frac{{\mathrm{ash in solid}}_{\%\mathrm{dry}}}{{\mathrm{ash in feedstock}\left(\mathrm{native}+\mathrm{inorganics}\right)}_{\%\mathrm{dry}}}$$

5 $${\mathrm{carbon yield}}_{\mathrm{product}}\left(\%\right)={\mathrm{mass yield}}_{\mathrm{product},\mathrm{dry}}\times \frac{{\mathrm{carbon in product}}_{\%\mathrm{dry}}}{{\mathrm{carbon in feedstock}}_{\%\mathrm{dry}}}$$

6 $${\mathrm{nitrogen yield}}_{\mathrm{product}}\left(\%\right)={\mathrm{mass yield}}_{\mathrm{product},\mathrm{dry}}\times \frac{{\mathrm{nitrogen in product}}_{\%\mathrm{dry}}}{{\mathrm{nitrogen in feedstock}}_{\%\mathrm{dry}}}$$

7 $$\mathrm{ER}\left(\%\right)=\frac{{\mathrm{mass}}_{\mathrm{product},\mathrm{dry}}}{{\mathrm{mass}}_{\mathrm{feedstock},\mathrm{dry}}}\times \frac{{\mathrm{HHV}}_{\mathrm{product},\mathrm{dry}}}{{\mathrm{HHV}}_{\mathrm{feedstock},\mathrm{dry}}}$$

### 2.5 Principal Component Analysis (PCA)

Principal component analysis (PCA) was performed to explore the complex data matrix and project it onto a reduced hyperspace defined by a new set of orthogonal variables known as principal components (PCs).^30^ These components were obtained as linear combinations of the original variables and were numbered in order of decreasing explained variance. In this work, PCA was conducted on three different data sets: GC-MS data of the biocrudes, GC-MS data of the aqueous phases, and the complete data set, using a toolbox developed in MATLAB by Ballabio.^30^ All three data sets were preprocessed via autoscaling, and the results were visualized in the two-dimensional space defined by the first two principal components (PC1–PC2).

## 3 Results and Discussion

### 3.1 Mass Yields

Figure 1 depicts the mass yields of biocrudes (A), solids (B) and gas (C) obtained with the different inorganics tested. To facilitate reading, the inorganics are grouped by anion type and arranged from left to right in increasing order of basicity (see Table 1). For alkali metals (Na and K), biocrude yield (Figure 1A) increased in the sequence: blank ∼ chlorides < sulfates < phosphates < carbonates ∼ oxides. This trend reflects the order of inorganic basicity and supports the hypothesis that higher basicity promotes biocrude production.^24^ This relationship was further explored by plotting biocrude and solid yields against inorganic basicity, expressed as the starting pH of the solution/suspension used for preparing the feedstock (Figure 2A-B). For both Na- and K-based inorganics, biocrude yields rose with basicity and peaked slightly at high pH with the carbonate form (Figure 2A). Compared with the blank, Na_2_CO_3_ and K_2_CO_3_ increased biocrude production by 47–48%, while Na_2_O and K_2_O achieved 39–50% increases. Similarly, Zhang et al. observed maximum yields at intermediate KOH concentrations during HTL of cotton stalk.^21^ Alkaline earth metals (Mg and Ca) showed smaller differences across anions compared with the blank (Figure 1A), though both displayed a slight maximum at high pH with carbonates (Figure 2A). CaCl_2_ stood out among the inorganics, as it was the only one to produce a significant decrease compared with the blank experiment (−31%).

**Figure 1:**
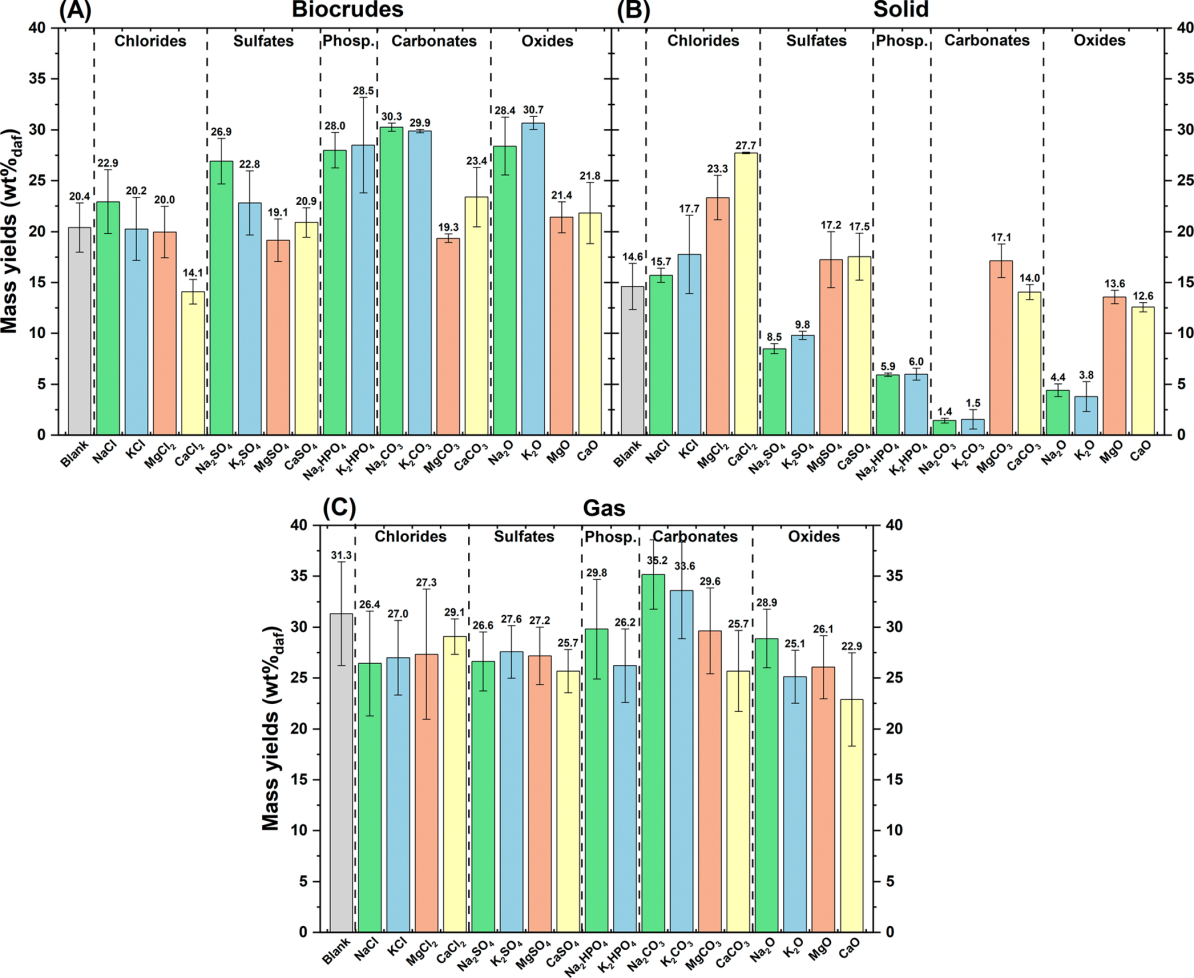
Mass yields for biocrudes (left), solid (center), and gas phase (right) on a dry and ash-free basis, according to Eq. 1. Error bars refer to the standard deviation of experiments performed at least in triplicate.

**Figure 2:**
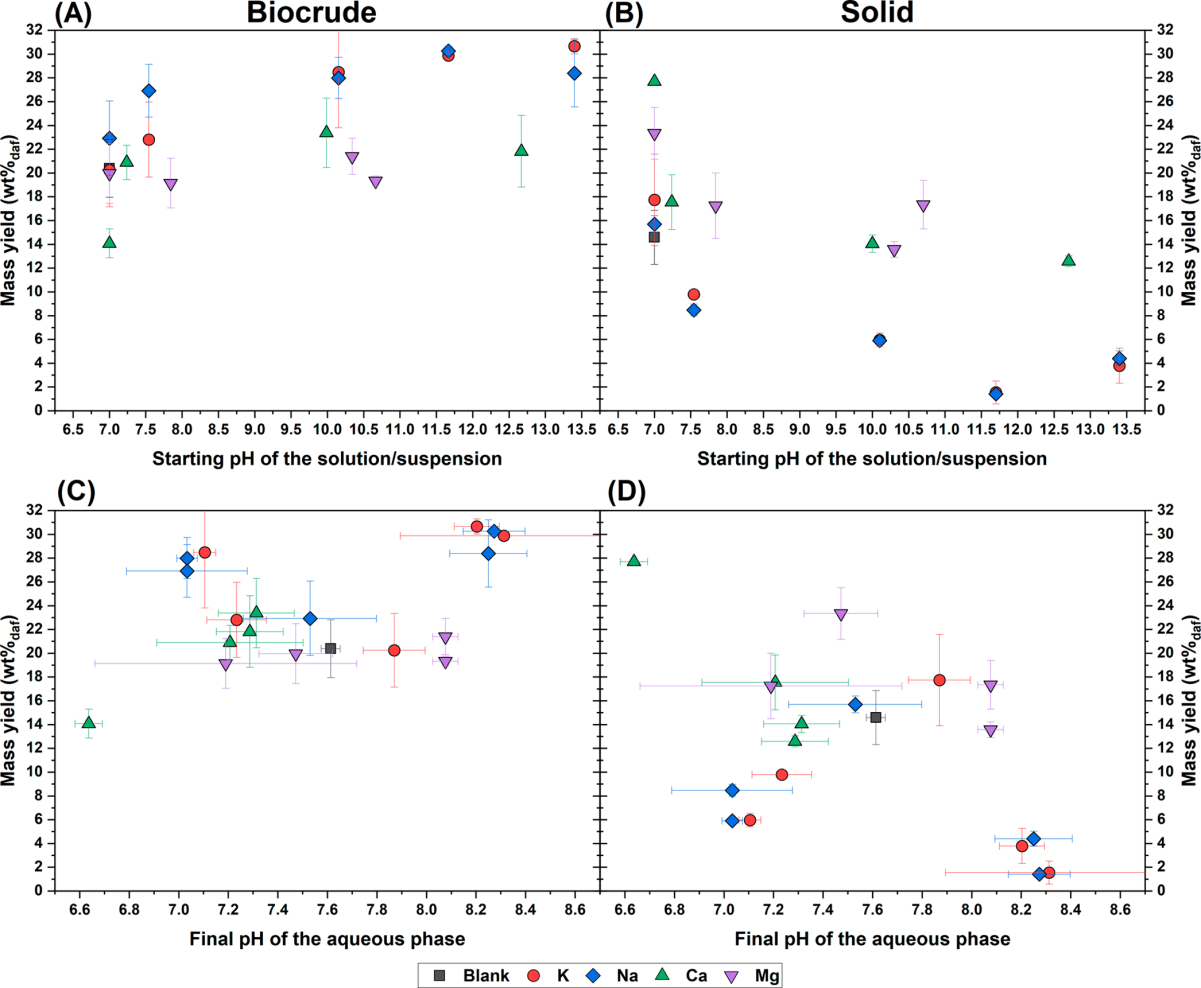
Mass yields of biocrude (A) and solid (B) plotted versus the starting pH of the solution, and the same mass yields of biocrude (C) and solid (D) plotted versus the final pH. Each symbol represents a single experimental condition, and error bars refer to the standard deviation of experiments performed in triplicate or more.

From the observation of solid yields (Figure 1B), a trend opposite to that of biocrude was observed. In fact, more basic inorganics generally led to reduced solid production, suggesting that higher basicity may promote the conversion of solid into biocrude. This aspect was examined more closely through Figure 2B, which clearly shows that, in complete contrast to biocrude, solid yields decreased with increasing basicity and reached a minimum at a high pH with the carbonate form. Excluding chlorides, which showed minimal variation, all alkali metal salts reduced solid formation, with reductions of up to 90% for Na_2_CO_3_ and K_2_CO_3_, and 70–74% for Na_2_O and K_2_O. Alkaline earth metals generally yielded similar or higher solid amounts compared with the blank, with the most pronounced increases observed for MgCl_2_ (+60%) and CaCl_2_ (+90%). Since numerous reactions take place during HTL, it is challenging to associate a specific phenomenon with the role of CaCl_2_. Solid formation is usually linked to condensation reactions, in which sugar dehydration plays a key role. As reported by Garcia-Sancho et al.,^31^ CaCl_2_ enhances 5-HMF yield, which under hydrothermal conditions serves as a precursor for solid humin formation. Interestingly, the authors highlighted that calcium cations, rather than chloride ions, have the major role. As suggested by Combs et al., Mg^2+^ shows higher selectivity toward levulinic acid, while Ca^2+^ is more prone to HMF formation.^32^ This observation is consistent with the results reported here for MgCl_2_, whose solid production was lower than CaCl_2_.

Regarding gas production (Figure 1C), the differences among the inorganics were limited, partly due to the high measurement uncertainty. Overall, the presence of inorganics tended to reduce gas yields, except for Na and K carbonates, which slightly increased gas production. A possible explanation is the formation of carbonates, as observed by He et al.^33^ Indeed, the gas phase generated from real waste and mixed-feedstock during HTL is typically rich in CO_2_,^28,34,35^ which originates in the liquid phase. Analysis of Ca-containing inorganics (Table S1) revealed that the CaO was converted into CaCO_3_, while only 11% of CaCl_2_ was transformed, and no CaCO_3_ was formed when using CaSO_4_. Although this behavior strongly depends on the relative solubility of the different inorganic species under hydrothermal conditions, it is likely that other metals also underwent partial carbonation. Theoretical calculations indicate that full carbonation of chlorides, sulfates, phosphates, and oxides could reduce the absolute gas yield by 2.2% daf for alkali metals and 4.4% daf for alkaline earth metals. While these values do not fully explain the differences observed in Figure 1C, carbonation likely occurs alongside other reactive influences, as seen for the solid and biocrude. He et al.^33^ also reported significant changes in gas composition and a decrease in gas yields during HTL of sewage sludge at 380 °C with CaO addition (minimum Ca/C = 0.5). These changes, in particular the reduction in CO_2_ and the increase in H_2_, were attributed to enhanced water–gas shift (WGS) activity. To evaluate this hypothesis, the experiment with CaO was repeated using a slightly different reactor setup and procedure, as previously described.^28^ The resulting gas composition is shown in Table S2. Given the dominant CO_2_ content and the limited presence of H_2_ and CO, it is unlikely that CaO significantly enhanced the WGS reaction in this case, possibly due to the lower Ca/C ratio (0.024).

Finally, it is worth noting that the mass balances do not close to 100% due to the presence of AP-solubles, whose mass cannot be reliably quantified due to their solubilization in the AP. These are commonly estimated by difference from 100%, assuming that water does not react with organic molecules. However, this assumption is well-known to be incorrect, as water participates in reactions such as hydrolysis and condensation,^36^ and it can lead to misleading interpretations. For this reason, in this work, AP-solubles were studied solely through carbon and nitrogen balances (Section 3.4).

### 3.2 pH Dependence

To better discuss the effect of pH on the HTL process, Figure 2 depicts the mass yields for solids and biocrudes as a function of pH. As partially commented above, each color in Figure 2A-B indicates that each cation follows a similar trend. Biocrude yields increase while solid yields decrease with increasing basicity of the coupled anion, reaching a maximum or minimum, respectively. However, without considering the cations (and thus the different colors in Figure 2A-B), no overall trend directly correlating performance with increasing basicity can be derived. Therefore, the initial inorganic basicity alone cannot fully explain the observed variations in phase distribution. Rather, these variations are largely influenced by the cation —with alkali metals yielding more biocrude and less solid than alkaline earth metals — and also by the anion, which mostly affects basicity.

Interestingly, a stronger overall correlation emerged when plotting mass yields against the final pH of the aqueous phase (Figure 2C–D). Excluding CaCl_2_, the data showed a strong maximum in solid yield and a minimum in biocrude yield at intermediate pH values. Notably, several inorganics, despite having a higher starting pH, resulted in a final pH lower than the blank experiment. As shown in Figure S3, the initial pH values (ranging from 7.0 to 13.4) converged postreaction to a narrower range (6.6–8.3), due to both increases in the final pH for less basic inorganics and decreases for more basic ones. This convergence is partially attributable to the low inorganic-to-feedstock ratio, which reduced the impact of the additives. Notably, the starting pH was measured before mixing with the feedstock, resulting in starting pH values of the feedstock slurry that were less dependent on the inorganics. In addition, an important contribution to pH variations likely arose from the changes in reaction pathways caused by the inorganics. The blank experiment, along with KCl, NaCl, and MgCl_2_, resulted in a slight increase in pH after the reaction (Figure S3), due to the greater formation of nitrogen-containing compounds from the protein fraction^37^ (see increased nitrogen yield in AP, Section 3.7). In contrast, the most basic inorganics caused a more pronounced drop in pH, likely due to higher production of AP-solubles (see carbon yield in AP, Section 3.7), as also observed by Zhu et al.^38^ Particularly noteworthy were Na_2_SO_4_, Na_2_HPO_4_, and K_2_HPO_4_, which not only significantly improved process performance (i.e., yielding higher biocrude and lower solid), but also led to final pH values even lower than that of the blank.

### 3.3 Elemental Composition

Figure 3A depicts the elemental compositions and HHVs of the biocrudes. In general, the presence of inorganics during HTL led to a decrease in HHV, with a minimum of 31 MJ/kg for Na_2_O compared with 35 MJ/kg in the blank experiment. In particular, oxides were the inorganics associated with the greatest reduction. This decrease in HHV is primarily due to a higher oxygen concentration when inorganics are present (13 wt % in the blank compared with a maximum of 21 wt % with Na_2_O). In the literature, the increasing or decreasing trend in HHV following the addition of inorganics has been observed to depend on the feedstock used.^17,24^ Specifically, more carbohydrate-rich feedstocks are more prone to decreasing their oxygen content and increasing their HHV after the addition of basic catalysts,^17,18,24^ while the opposite trend has been observed for other feedstocks.^22,24^ Given the considerable amount of lipids present in the feedstock used in this work, a large fraction of the biocrude was composed of fatty acids, as will be presented in Section 3.5. Consequently, the increased biocrude yield with inorganics can be attributed to the formation of additional molecules that raise the overall oxygen concentration. Additionally, another HHV trend emerged based on the metal cation, as further discussed in Section 3.7 using PCA. In all cases, Na-containing inorganics resulted in a lower HHV than K-containing counterparts, while Mg-containing inorganics produced a lower HHV than Ca-containing ones. This finding is particularly noteworthy for the two alkali metals, as their mass yields did not differ significantly, suggesting a direct cation-specific effect on biocrude quality.

**Figure 3:**
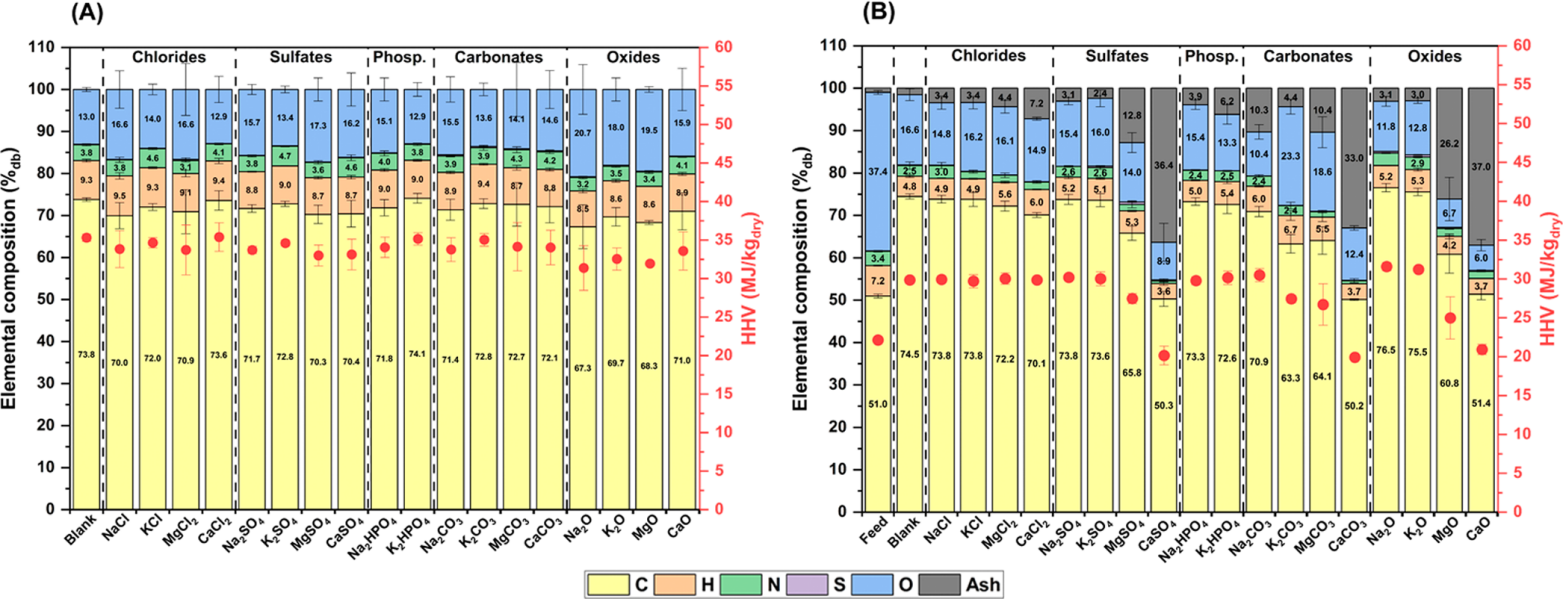
Elemental composition (bars) and HHVs (red dots) of biocrudes (A) and solids (B). Error bars refer to the standard deviation of experiments performed at least in triplicate.

Figure 3B depicts the elemental compositions and HHVs of the solids. Compared with the feedstock, the solid produced in the blank experiment exhibited a higher carbon concentration and a lower concentration of oxygen, nitrogen, and hydrogen, resulting in a higher energy density. Relative to the blank experiment, the presence of most inorganics during HTL did not significantly affect the carbon content (∼73 wt %) or the HHV (∼30 MJ/kg). Exceptions included K_2_CO_3_, which showed a high oxygen content and yielded very little solid (Figure 1B), as well as all alkaline earth metals except the chlorides. When not present as chlorides, alkaline earth metals exhibited lower water solubility, resulting in significantly higher ash contents in the solids and, consequently, reduced HHV values. In contrast, the greatest increases in carbon content and HHV were observed with Na_2_O and K_2_O, reaching 76–77 wt % and 31–32 MJ/kg, respectively.

The different solubilities of the inorganics can be observed in Figure S4, which depicts the ash yield in the final solids. Generally, Ca- and Mg-containing inorganics resulted in a higher retention in the final solids compared with K- and Na-containing inorganics. Notably, CaO exhibited a final ash content in the solid that exceeded the amount of inorganic initially added, further confirming its conversion to CaCO_3_. K and Na exhibited very limited retention in the solids, with ash yield consistently below 8%, suggesting their almost complete presence in the aqueous phase. This aspect must be taken into consideration when planning the possible recirculation of the aqueous phase to avoid the accumulation of inorganics and potential antagonistic effects.^39^

To better understand the variations in elemental composition, the H/C, O/C, and N/C molar ratios of solids and biocrudes are reported in Figure 4. Compared with the feedstock, biocrude samples generally exhibited more than a 2-fold reduction in O/C (Figure 4A), a moderate decrease in N/C (Figure 4B), and a slight decrease in H/C. When compared with linoleic acid (H/C = 1.78; O/C = 0.11; N/C = 0.00), a primary product expected from the hydrolysis of sunflower oil, the biocrude from the blank experiment showed a slightly lower H/C (1.51), a similar O/C (0.13), and a significantly higher N/C (0.044). This observation corroborates the incorporation in the biocrudes of compounds beyond fatty acids, characterized by greater unsaturation (lower H/C), comparable oxygen content, and embedded nitrogen. This aspect will be further explored using GC-MS analysis in Section 3.5.

**Figure 4:**
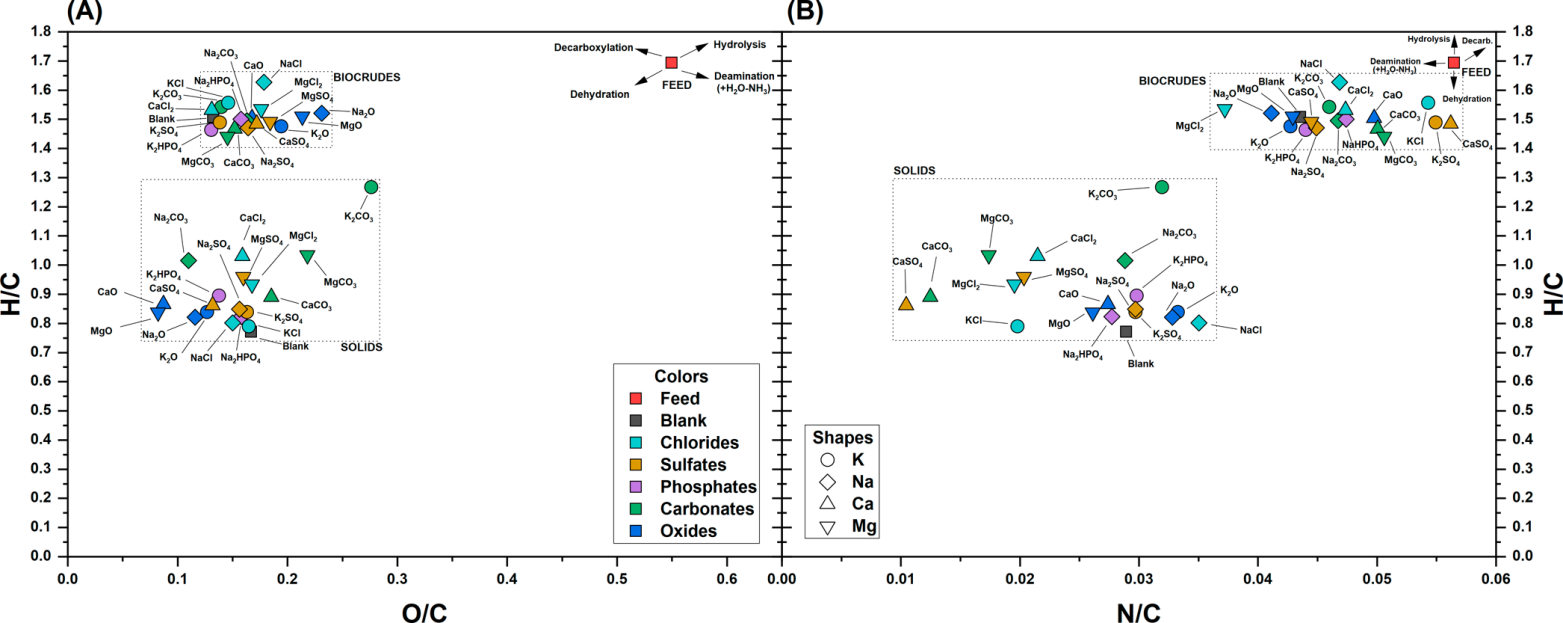
Van Krevelen diagrams for solids and biocrudes. (A) H/C vs O/C; (B) H/C vs N/C. The shape of the symbols refers to the cation, while the color refers to the anion, as shown in the legends.

Interestingly, the O/C ratio in the blank experiment (0.13) was lower than in the samples tested with inorganics, with the highest values registered with oxides (0.23 with Na_2_O). The N/C ratios varied more widely (0.037–0.056); however, except for oxides, most biocrudes produced with inorganics exhibited N/C values between those of the feedstock and the blank. The increased O/C and N/C ratios in the presence of inorganics must hence be attributed to the formation of apolar compounds enriched in oxygen and nitrogen, which also led to higher biocrude yields. A closer examination of Figure 4 may reveal compositional trends across different groups, which will be further analyzed through PCA in Section 3.7.

Focusing on the solids, the H/C, O/C, and N/C elemental ratios decreased significantly compared with the feed. These changes can be attributed primarily to dehydration reactions, as clearly indicated by the arrows in Figure 4A-B, and to deamination reactions as shown by the arrow in Figure 4B. It should be noted that, due to the lower abundance of nitrogen relative to oxygen, the impact of deamination reactions is almost imperceptible in Figure 4A. Even excluding the sample obtained with K_2_CO_3_, whose extreme values can be justified by the limited amount of solid produced, the range of elemental ratios was quite broad: O/C = 0.08–0.22, H/C = 0.79–1.03, and N/C = 0.010–0.035. The blank sample exhibited a lower H/C ratio than the samples obtained in the presence of inorganics. Notably, a trend in the extent of the dehydration reaction could be observed in Figure 4A, following the order: oxides > chlorides ∼ sulfates ∼ phosphates ∼ blank > carbonates. Although dehydration is well-known to be favored under acidic conditions,^40^ this trend did not correlate with the measured pH. Furthermore, the dehydration trend differed from that of the solid yields shown in Figure 1, suggesting the involvement of additional chemical reactions beyond dehydration and deamination.

### 3.4 Carbon, Nitrogen and Energy Yields

Figure 5A depicts the carbon yields across all the obtained phases. Except for MgCl_2_ and CaCl_2_, all experiments resulted in the highest proportion of carbon being present in the biocrudes. Since the differences in mass yields were consistently greater (Figure 1) than the differences in carbon content of the biocrudes (Figure 3), the trends in carbon yields resembled those observed in mass yields. Notably, with K_2_O, Na_2_CO_3_, and K_2_CO_3_, up to 42% of the carbon in the feedstock was converted into biocrude, significantly higher than the 29% achieved in the blank experiment.

**Figure 5:**
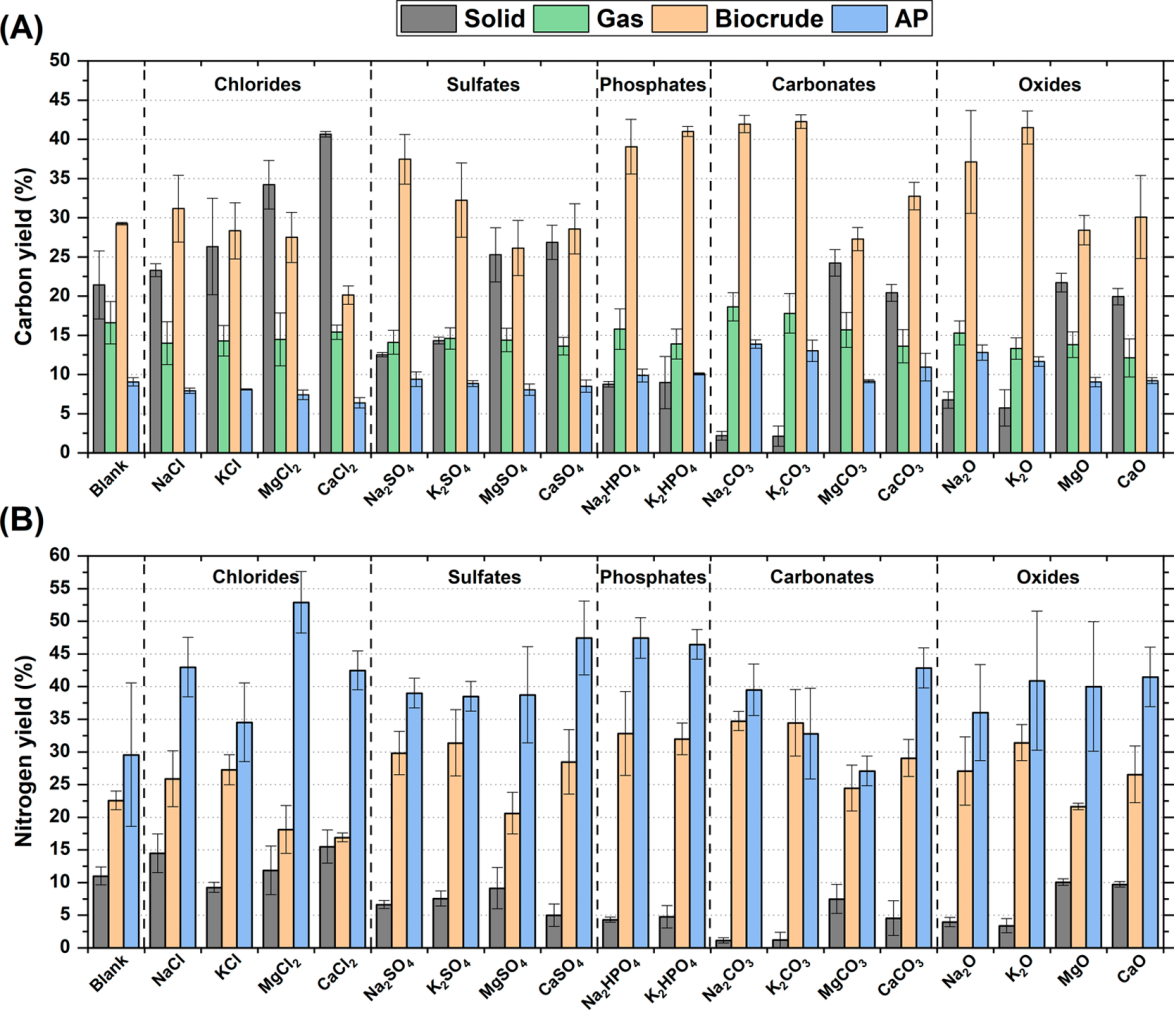
Carbon (A) and nitrogen (B) yields across the different phases. Error bars refer to the standard deviation of experiments performed at least in triplicate.

As with the biocrude, the trends in carbon yields for the solid and gas phases closely mirrored those of their respective mass yields. The variation in carbon content on a daf basis was minimal among the solid samples, while the carbon yield in the gas phase was calculated under the assumption that it consisted solely of CO_2_, as experimentally confirmed (Table S2). The solid carbon yield varied significantly, ranging from a maximum of 41% with CaCl_2_ to a minimum of only 2% with Na_2_CO_3_ and K_2_CO_3_. The gas carbon yield was relatively consistent across the different tests (12–19%), while the carbon yield in the aqueous phase was comparatively low, ranging from 6 to 14%.

Most of the nitrogen was distributed in the aqueous phase, with yields ranging from 27% to 53% (Figure 5B). This trend can be attributed to the high polarity of nitrogen-containing compounds and their strong affinity for water. Interestingly, despite the large standard deviation observed for the blank experiment, it generally resulted in a lower nitrogen yield compared with most of the inorganics tested. The biocrude phase accounted for the second-highest fraction of nitrogen (17–35%), driven by both its elevated N/C ratio (Figure 4B) and higher mass yields relative to the solid phase. Consequently, nitrogen yields in the solid phase were the lowest, ranging from only 1% to 15%.

It is worth noting that the overall carbon and nitrogen balances averaged approximately 75%, with some variation across experiments, ranging from 70 to 84% for carbon and 59–84% for nitrogen. These values are in line with those reported in previous studies,^9,15,28^ and are likely influenced by losses during phase separation.

Figure 6 depicts the energy recovery in solids and biocrudes for the various tests performed. For the biocrudes, the trends among the different inorganics tested were similar to those observed for carbon yields, with a maximum of 45–46% of the feedstock energy transferred into the biocrude in the cases of Na_2_CO_3_ and K_2_CO_3_. Although the overall trend with varying inorganics resembled that of carbon yields, a slight difference emerged with respect to the blank experiment. Specifically, the increase in energy recovery in the presence of inorganics was relatively smaller than the increase previously observed in mass yields. Besides CaCl_2_, other inorganics such as MgSO_4_, MgCO_3_, and MgO also resulted in lower energy recovery compared with the blank experiment. This reduction in energy recovery was attributed to the lower energy density of the biocrudes, as described in Section 3.3.

**Figure 6:**
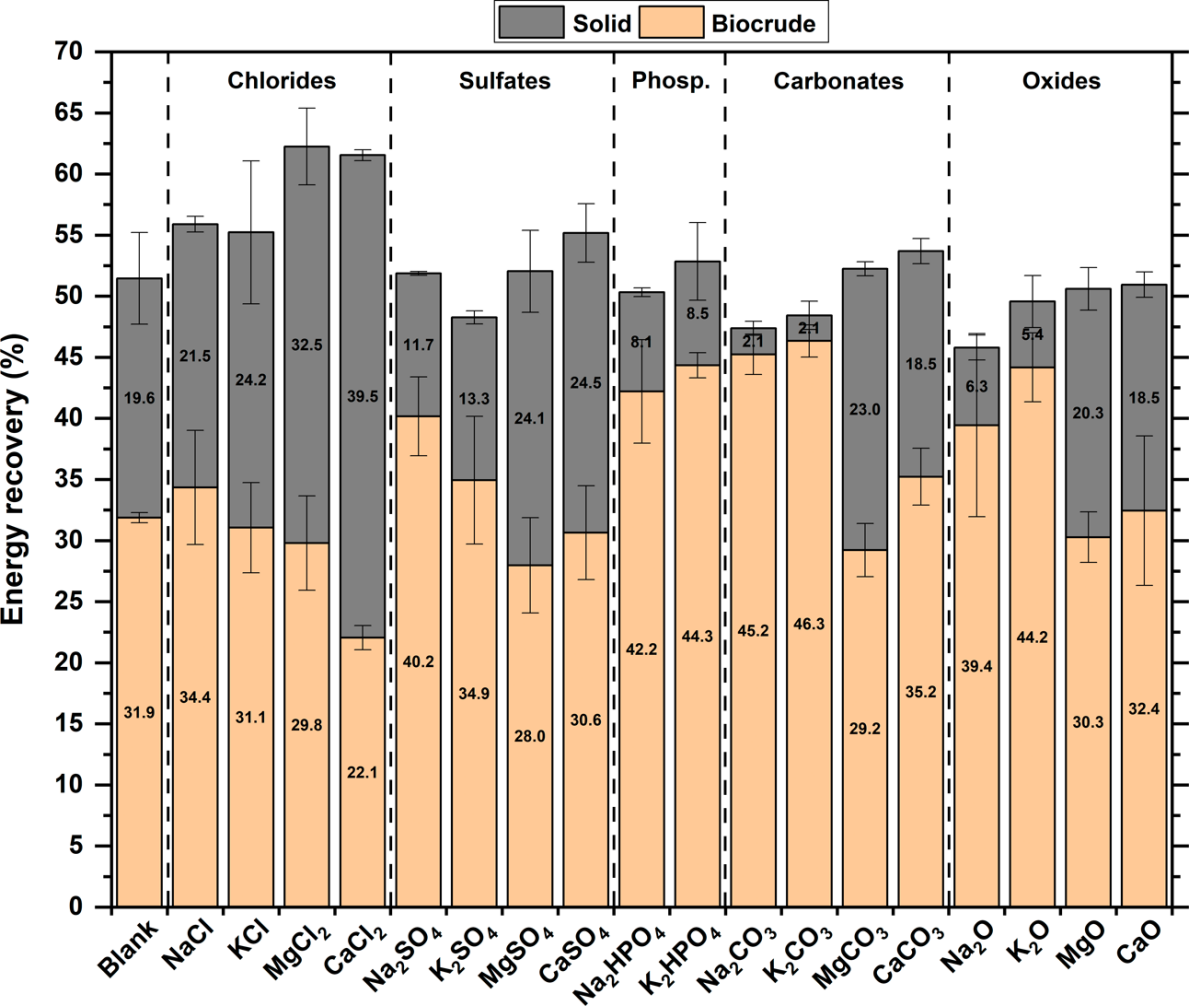
Energy recoveries as biocrude (orange) and solid (gray). Error bars refer to the standard deviation of experiments performed at least in triplicate.

Energy recovery in the residual solid varied greatly depending on the inorganic tested, with a minimum of 2% recorded with Na_2_CO_3_ and K_2_CO_3_, and a maximum of 39% observed with CaCl_2_. Interestingly, an inverse relationship was noted between energy recovery in the solid and in the biocrude, with the total energy recovery being slightly lower in cases with reduced solid recovery (Figure S5). Specifically, the combined energy recovery from solid and biocrude was 62% with MgCl_2_ and CaCl_2_, but only 47–48% with Na_2_CO_3_ and K_2_CO_3_. This trend may be attributed to losses during the conversion of produced solid into further biocrude, as already observed in previous works,^34^ preventing complete energy transfer.

### 3.5 Biocrude Characterization

The composition of the biocrudes resulting from GC-MS analysis is reported in Figure 7. The majority of the compounds identified (>70%) were long-chain fatty acids. These primarily derive from triglyceride hydrolysis, which accounted for 18% of the feedstock used in the tests. Additionally, it is important to consider that GC-MS analysis captures only the lighter fraction of the biocrude, which includes C18 fatty acids. These two factors together explain the predominance of fatty acids from GC-MS analysis and help account for the limited observable differences between the tests. In fact, no significant variation was detected among the tests, except in those involving MgCl_2_ and CaCl_2_. The tests with CaCl_2_ resulted in the lowest recorded amount of fatty acids, suggesting reduced triglyceride hydrolysis, consistent with its lower biocrude production (Figure 1).

**Figure 7:**
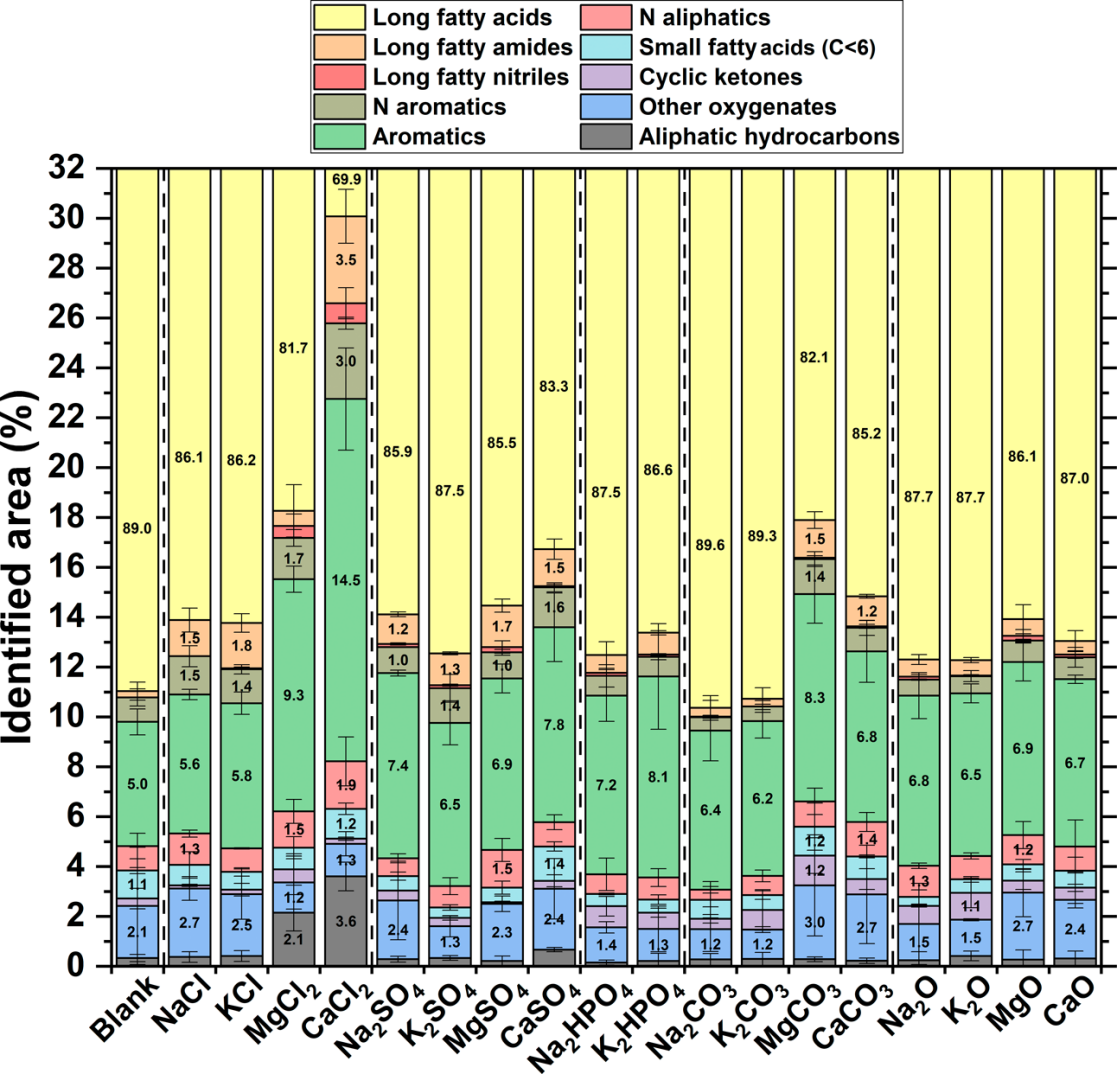
Relative abundance of different classes of compounds in the biocrudes from GC-MS analysis. Priority in the nomenclature process follows the descending order indicated in the legend, from “long fatty acids” to “aliphatic hydrocarbons”. “Other oxygenates” refers to alcohols, esters, linear ketones, and ethers. Error bars refer to the standard deviation of measurements performed at least in duplicate.

In all runs, the second most common group of molecules was aromatics, mostly phenols, with only a very small fraction attributed to furan-derived structures. All other molecular groups were present in much lower amounts. Notably, CaCl_2_ and MgCl_2_ led to significantly higher levels of both saturated and unsaturated aliphatic hydrocarbons. The majority of these corresponded to C17 linear hydrocarbons, which may result from the decarboxylation of fatty acids. For instance, the most intense peak was 8-heptadecene, a known product of oleic acid decarboxylation. This suggests that CaCl_2_ and MgCl_2_ may catalyze the decarboxylation of fatty acids, in accordance with the increased decarboxylation observed by Kang et al. during hydrothermal treatment of oil shale after addition of CaCl_2_.^41^ However, this effect appears to be limited with the concentrations tested in this work, as indicated by the still low aliphatic hydrocarbon content in the biocrudes and the insignificant increase in gas-phase production shown in Figure 1C.

From a more analytical perspective, Figure S6 shows the PCA results based on the data presented in Figure 7. Excluding CaCl_2_ and MgCl_2_, the data can be grouped into two clusters. The first cluster comprised the most basic Na- and K-containing inorganics (Na_2_O, K_2_O, Na_2_CO_3_, K_2_CO_3_, Na_2_HPO_4_, K_2_HPO_4_), while the second included most of the Ca- and Mg-containing inorganics (MgO, CaO, MgCO_3_, CaCO_3_, MgSO_4_, CaSO_4_), along with the blank experiment and the Na and K chlorides. The first cluster was characterized by a higher fraction of cyclic ketones, long-chain fatty nitriles, aromatics, and aliphatic hydrocarbons, and a lower proportion of other oxygenates, short-chain fatty acids, and nitrogen-containing aliphatics and aromatics. The opposite trend was observed in the second cluster. Specifically, Figure 7 shows that Ca and Mg promoted the formation of “other oxygenates” (alcohols, esters, linear ketones, ethers), which explains most of the differentiation in the PCA between the two clusters. However, it should be emphasized once again that the PCA amplified minor differences present in the GC-MS data, due to the dominant presence of fatty acids. As a result, the different biocrudes cannot be considered significantly distinct.

The FTIR spectrum of the biocrude obtained from the blank experiment is shown in Figure 8 and closely resembled the spectra from all tests (Figure S7). The broad peak between 3600–2500 cm^–1^ corresponded to the O–H stretching vibration of carboxylic acids. In the range of 2954–2851 cm^–1^, three peaks were associated with saturated C–H stretching, while smaller peaks at 1456, 1437, and 1377 cm^–1^ corresponded to their bending vibrations. The strong peak at 1705 cm^–1^ was attributed to aliphatic C = O stretching, which, together with the O–H stretching, indicated a high content of fatty acids in the biocrude, as confirmed by the GC-MS analysis (Figure 7). Additional, less intense peaks at 1614, 1410, 966, and 723 cm^–1^ indicated the presence of unsaturated structures, either within aromatic rings or as isolated double bonds. The broad region between 1296–1205 cm^–1^ was attributed to C–O stretching, likely from phenols and ethers.

**Figure 8:**
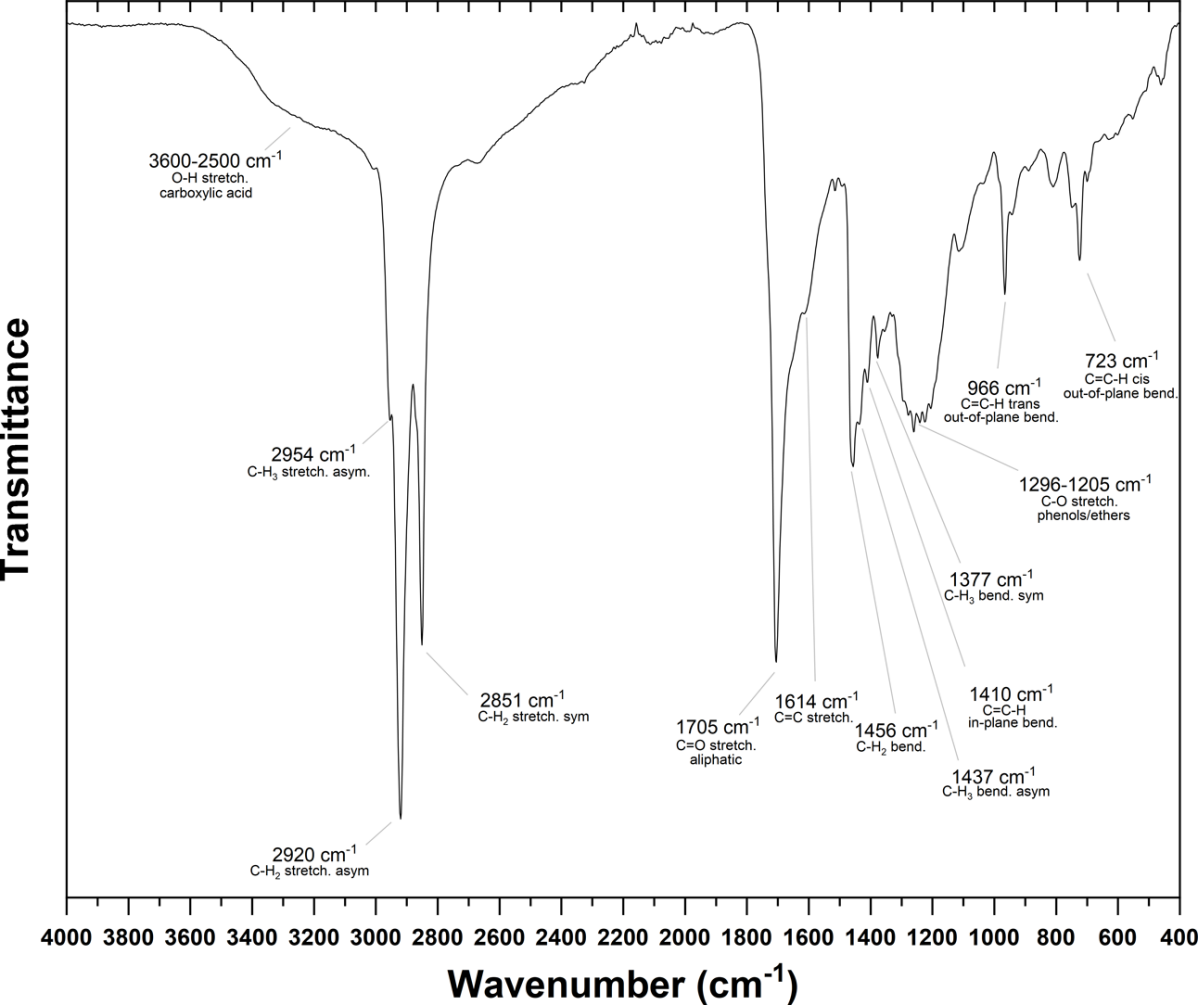
ATR-FTIR of the biocrude obtained from the blank experiment. Most intense peaks are associated with the corresponding vibrations.

The FTIR spectra from all tests closely resembled that shown in Figure 8. The only noticeable differences were the presence of two distinct peaks at 1219 cm^–1^ and 520 cm^–1^, which were more prominent with Na_2_O, K_2_O, and MgO, and slightly visible with Na_2_CO_3_ and K_2_CO_3_ (Figure S7). This strong similarity further confirmed that, although the quantity of biocrude produced varied significantly, its quality was largely unaffected by the type of inorganics present.

Figure 9 presents a preliminary evaluation of the distillation cuts of the biocrudes obtained via TGA for three experiments: the blank, CaCl_2_, and K_2_CO_3_. The blank experiment served as a reference, while CaCl_2_ and K_2_CO_3_ represented the minimum and one of the maximum values in biocrude production, respectively (Figure 1A). Figure 9A shows the distribution across different boiling ranges, normalized per gram of biocrude. Overall, the differences among the three samples were minimal. Most of each biocrude (73–77%) boiled below 400 °C, confirming the potential for distillate recovery, primarily kerosene and diesel. In contrast, only a smaller portion (11–15%) consisted of nonvolatile matter. The only notable distinctions were a slight shift toward lower boiling points for K_2_CO_3_, shown by a decrease in the 200–300 °C range in favor of the 25–200 °C range, and a slight increase in nonvolatile matter for CaCl_2_. These differences, however, are relatively small compared with the substantial variation in overall biocrude yields (blank: 20.4%, CaCl_2_: 14.1%, K_2_CO_3_: 29.9%). For this reason, examining the mass yield of each distillation cut relative to the feedstock (Figure 9B), all cuts followed the trend: K_2_CO_3_ > blank > CaCl_2_. Together with the complementary analyses discussed above, these results indicate that variations in biocrude yields in the presence of inorganics were not due to a shift in reaction selectivity, but rather to lower solid formation, which allowed greater biocrude production without substantially altering its properties.

**Figure 9:**
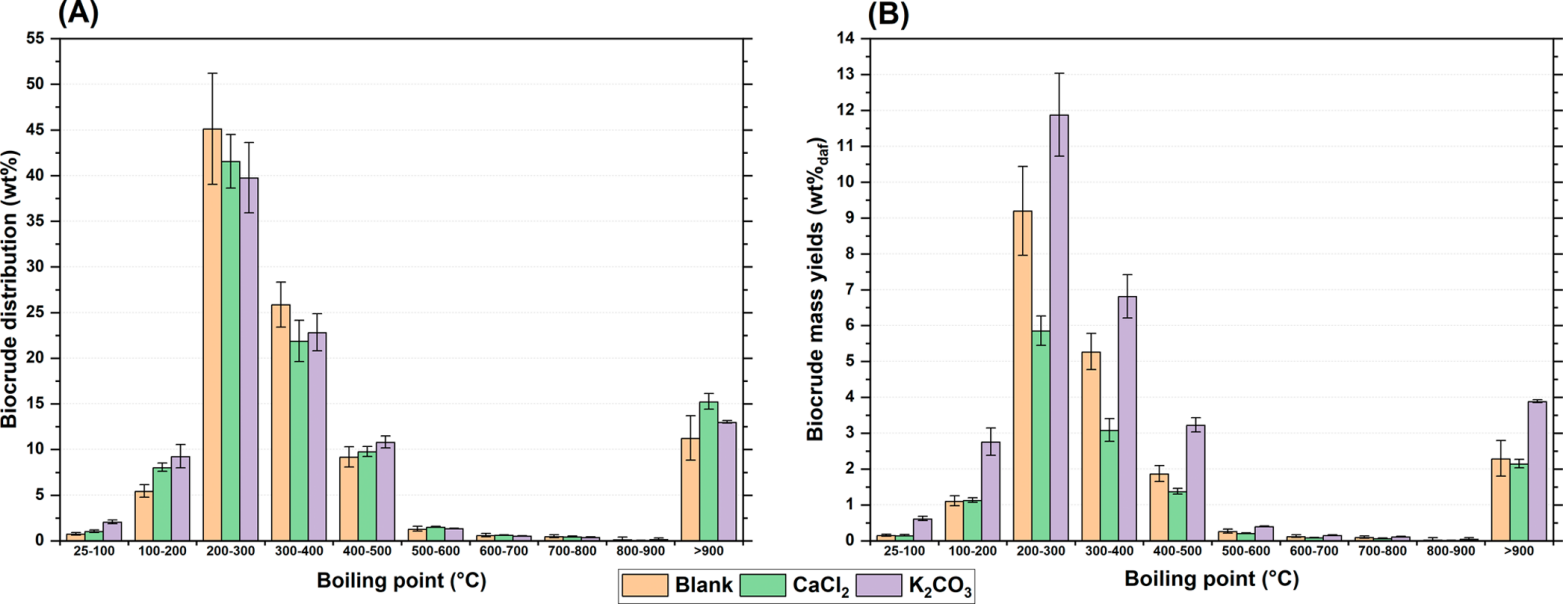
(A) Boiling point distribution of the different boiling ranges obtained from TGA in argon. The values are expressed as grams boiling in a specific temperature range per gram of biocrude. (B) Mass yields of each boiling range relative to the starting feedstock (g boiling in a certain temperature range/g dry and ash-free feedstock). The “>900” columns indicate the mass loss after switching to an air flow. Error bars refer to the standard deviation of measurements performed at least in duplicate.

### 3.6 Aqueous Phase Composition

The composition of the aqueous phase (photograph in Figure S8) was evaluated by GC-MS without any derivatization, and the detected compounds were grouped into the families depicted in Figure 10. To provide a more robust interpretation, a PCA of the same data set is reported in Figure 11. The most abundant family of molecules consisted of carboxylic acids, including acetic, propanoic, butanoic, and various methyl-substituted acids. Acetic acid was the predominant carboxylic acid, as it is recognized as the final product of various degradation pathways and the most stable carboxylic acid.^42,43^ Figure 11A reveals that lower PC1 values correspond primarily to samples with higher carboxylic acid content, and Figure 11B illustrates a PC1 trend following the order: chlorides ∼ phosphates < sulfates ∼ blank < oxides ∼ carbonates (Figure 11A). This trend is further supported by a detailed inspection of Figure 10. Additionally, an inverse relationship between carboxylic acid concentration and the pH of the aqueous phase (Figure S3) reinforces the correlation between carboxylic acid formation and increased acidity.

**Figure 10:**
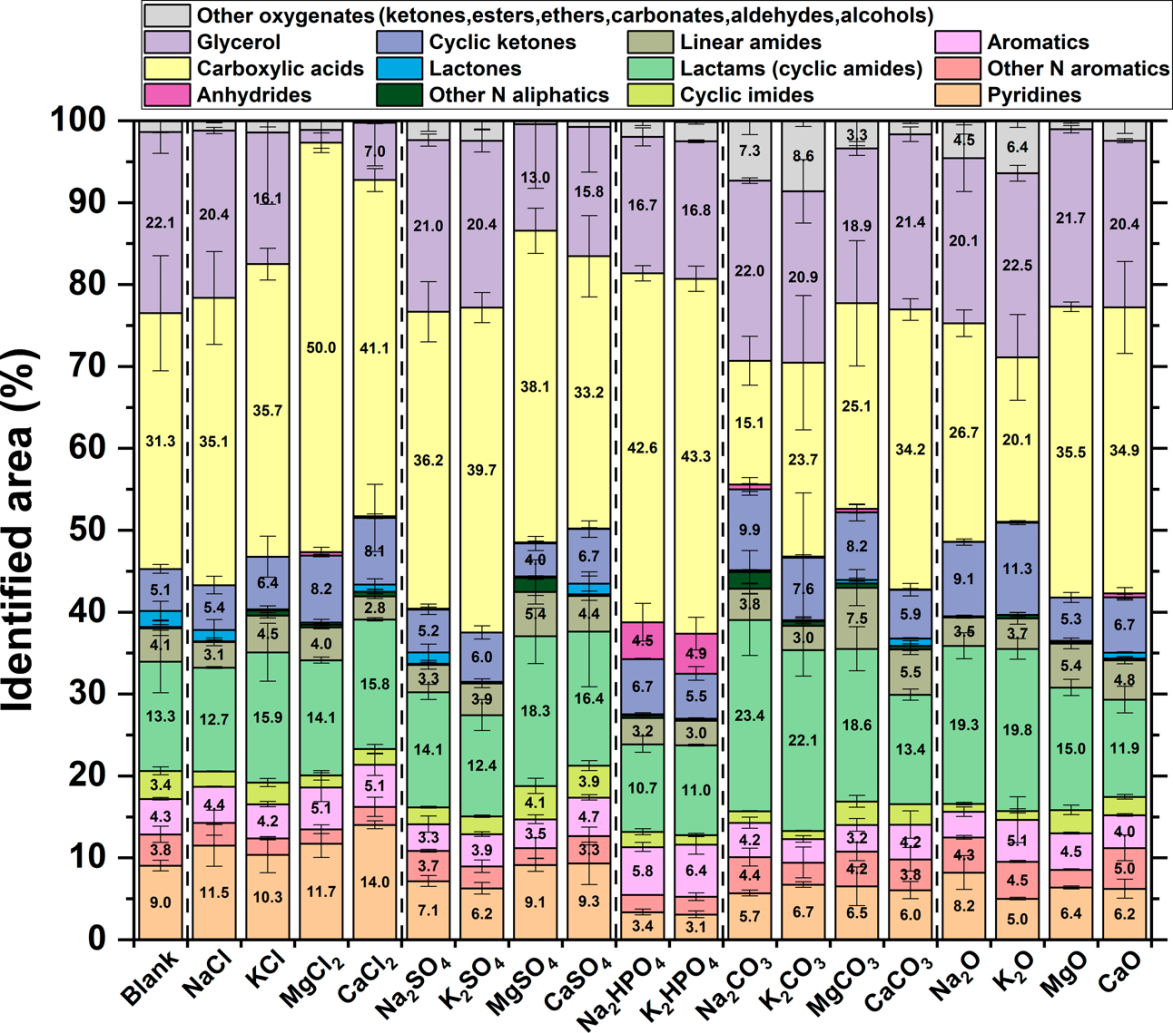
Relative abundance of different classes of compounds in the AP from GC-MS analysis. Priority in the nomenclature process follows the ascending order indicated in the legend, from “pyridines” to “other oxygenates”. Error bars refer to the standard deviation of measurements performed at least in duplicate.

**Figure 11:**
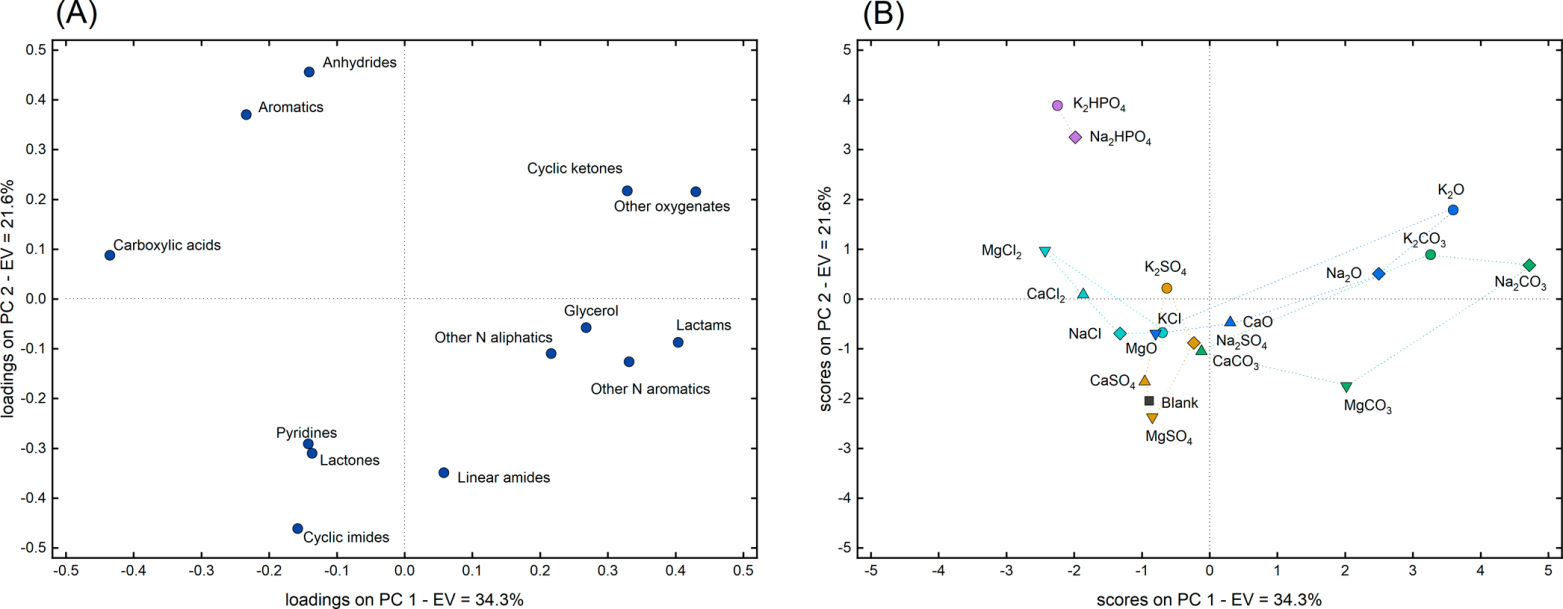
Loadings (A) and scores (B) obtained from the principal component analysis (PCA) of the compound families shown in Figure 10. EV denotes the explained variance of each principal component (PC) shown. The symbols used for the inorganic compounds in the scores plot are subdivided according to their cations and anions. Anion subdivision is based on color: oxides (blue), carbonates (green), hydrogen phosphates (purple), sulfates (mustard yellow), and chlorides (light blue). Cation subdivision is based on shape: K (circle), Na (diamond), Ca (upward-pointing triangle), and Mg (downward-pointing triangle).

Glycerol was the second most abundant compound and remained nearly constant across the different tests, except in the presence of CaCl_2_ and MgCl_2_, as also observed for the concentration of long-chain fatty acids in the biocrudes (Figure 7). Glycerol is the coproduct of hydrolysis of triglycerides, along with fatty acids, and showed high stability under hydrothermal environment.^9^ The amount of lactams, especially pyrrolidinones, was significant, and their trend in Figure 11A was opposite to that of carboxylic acids, suggesting increased production at higher pH. Pyrrolidinones were observed to derive from glutamic acid via internal cyclization, forming an amide bond between the side-chain carboxylic acid and the amino group.^11^ Amide formation under hydrothermal environment has been observed to be pH dependent.^44,45^ Hence, the presence of different inorganics could effectively influence the reaction equilibrium. Linear amides, primarily acetamide and *N*-methyl acetamide, likely formed from the reactions of acetic acid with ammonia and methylamine, respectively.^22^ These compounds did not follow the same trend as lactams, despite both requiring amide bond formation. This discrepancy may be attributed to the fact that acetamide formation depends on the presence of acetic acid, whose production in this work was favored by less basic inorganics. Pyridines were also identified in the aqueous phase, likely due to their sufficient polarity. Their formation was particularly enhanced in the presence of chlorides and reduced in the presence of phosphates. The increased abundance of pyridines and other nitrogen-containing aromatics in the aqueous phase, along with the higher concentration of N-containing aromatics in the biocrudes (Figure 7), suggests a potential catalytic effect of chlorides on this class of molecules. Similarly, Lin et al. observed a significant increase in N-containing heterocycles upon the addition of NaCl during the HTL of proteins.^46^

Phosphates led to the formation of butanoic anhydride, a compound not detected with the other inorganics, and to a higher abundance of aromatics, including phenols, hydrocinnamic acid, and benzoic acid, as highlighted in Figure 11. On the other hand, Na_2_CO_3_, K_2_CO_3_, Na_2_O, K_2_O favored the production of cyclic ketones and other oxygenated compounds, predominantly alcohols such as propylene glycol, propanediol, and tetrahydrofurfuryl alcohol.

### 3.7 PCA

Given the large amount of data obtained from the various tests, a PCA of the complete data set was conducted to highlight any possible trends. The GC–MS data were excluded from this analysis, as they were evaluated separately through dedicated PCAs. Figure 12A shows the distribution of the measured variables in the PC1–PC2 space (loadings), while Figure 12B illustrates the positioning of the different tests within the same space (scores).

**Figure 12:**
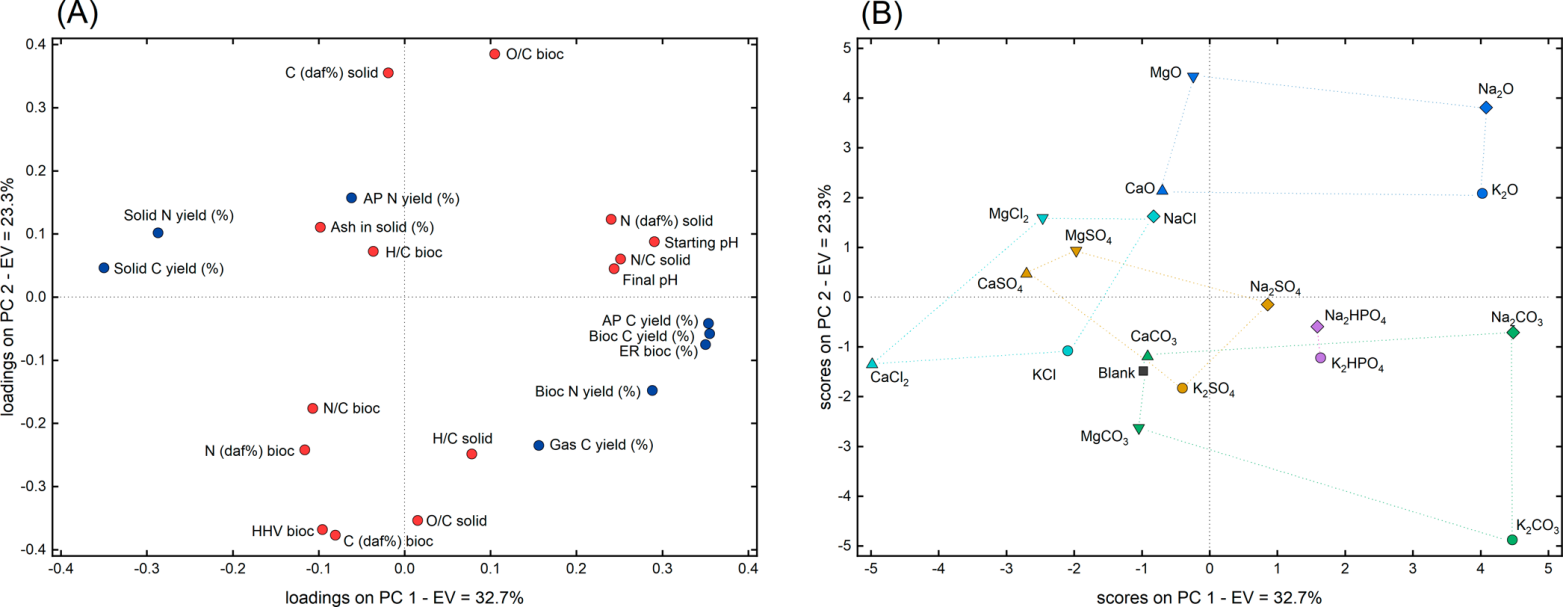
Loadings (A) and scores (B) obtained from the principal component analysis (PCA) of the complete data set. EV denotes the explained variance of each principal component (PC) shown. The color of the circles used for the loadings indicates whether they are yield-related (blue) or property-related (red). The symbols used for the inorganic compounds in the scores plot are subdivided according to their cations and anions. Anion subdivision is based on color: oxides (blue), carbonates (green), hydrogen phosphates (purple), sulfates (mustard yellow), and chlorides (light blue). Cation subdivision is based on shape: K (circle), Na (diamond), Ca (upward-pointing triangle), and Mg (downward-pointing triangle).

Variables associated with yields (blue dots in Figure 12A), including carbon and nitrogen yields, as well as energy recovery, were primarily distributed along PC1. In contrast, variables related to product properties (red dots in Figure 12A), such as elemental compositions and ratios, ash content, HHV, and pH, were mainly distributed along PC2. Specifically, higher PC1 values, associated with higher pH, favored carbon distribution toward the AP and biocrude (as well as higher ER) at the expense of the solid fraction, and favored nitrogen yield in the biocrude at the expense of those in the solid and AP fractions. Simultaneously, lower PC2 values were linked to improved biocrude properties, namely higher HHV, higher carbon content, and lower O/C ratio. Interestingly, the N/C showed behavior opposite to that of O/C, making their simultaneous reduction impossible.

Focusing on the distribution of the performed tests (Figure 12B), the arrangement along PC1 mirrored the carbon yields in biocrudes and solids (Figure 5). In fact, higher PC1 values corresponded to increased biocrude yields and decreased solid yields, as also reflected in Figure 12A. Even more notable is the presence of a consistent pattern within each anion group. Specifically, with the only exception of the MgCO_3_/CaCO_3_ couple, the four metal cations were consistently distributed in a rectangular arrangement, with Na and K at higher PC1 values (with Ca and Mg at lower PC1 values), and Na and Mg at higher PC2 values (with K and Ca at lower PC2 values). This recurring trend suggests that the metal cations had a coherent and direct effect on performance: Na and K promoted biocrude production and solid conversion more effectively than Mg and Ca, while K and Ca generally resulted in biocrude with lower oxygen content and higher energy density. The higher activity of alkali metals (Na and K) compared with alkaline-earth metals (Mg and Ca) may partly be attributed to their generally higher solubility. However, solubility data near the critical point of water remain scarce, making it difficult to determine whether the salts acted primarily through homogeneous or heterogeneous mechanisms under the conditions applied. On the other hand, the performance difference between Na and K is particularly significant, as their salts do not differ substantially in solubility or basicity. This suggests that the superior performance of K relative to Na may be linked to intrinsic differences in the catalytic activity of the metal cations.

## 4 Conclusions

The effects of the four most common metals—Na, K, Mg, and Ca—introduced as oxides, carbonates, phosphates, sulfates, and chlorides were evaluated during the hydrothermal liquefaction (HTL) of a synthetic representative food waste. Overall, the presence of inorganics markedly influenced HTL performance. The most notable effect was observed in product distribution, with biocrude production favored at the expense of solid in the presence of inorganics.

Focusing on the effect of anions, the enhancement in biocrude yield followed the order: blank ∼ chlorides < sulfates < phosphates < carbonates ∼ oxides, with a slight maximum in correspondence of carbonates. This trend approximately followed increasing basicity, except for carbonates, which produced slightly more biocrude than oxides despite being less basic. This observation suggests that the influence of inorganics may be mostly related to their basicity, as commonly stated in the literature. However, this relationship between HTL performance and inorganic basicity was observed only when varying the anion while keeping the metal cation constant. No clear correlation emerged when plotting the initial basicity of all tested inorganics against solid and biocrude mass yields. Therefore, basicity alone cannot fully explain the observed performance variations, indicating an additional contribution from the metal cations. Specifically, with respect to cations, the trend in biocrude yield was: Ca ∼ Mg ≪ K ∼ Na.

In terms of biocrude properties, variations were observed in elemental composition. Specifically, the presence of inorganics promoted the incorporation of more recalcitrant, heteroatom-rich molecules, which slightly reduced the energy density of the biocrude. Notably, K generally produced biocrude with superior elemental properties compared with Na. As the same molar amount of metal was used in all experiments, this difference cannot be attributed to variations in anion concentration or initial basicity, supporting the hypothesis of a direct catalytic effect of the metal cations. GC-MS, FTIR, and TGA analyses of the biocrudes showed negligible compositional differences among the tests, suggesting that the primary impact of inorganics was to limit solid production through mechanisms already present under inorganic-free conditions, without significantly altering biocrude selectivity. GC-MS analysis of the aqueous phase did indicate some variations in reaction mechanisms; however, these were less pronounced than the overall enhancement of existing pathways.

Overall, this study highlights the critical role of inorganics in HTL, demonstrating that their influence in organic waste processing cannot be overlooked and providing a solid framework for understanding how different inorganic species affect process performance.

## Supplementary Material



## Notes

Literature survey of the biochemical composition of food waste and OFMSW (Figure S1); temperature profile inside the reactor during the reaction (Figure S2); CaCO_3_ concentration and yield in final ash (Table S1); gas composition obtained from HTL test using CaO as inorganic (Table S2); final pH of the aqueous phase (Figure S3); ash content in the produced solid (Figure S4); energy recoveries of biocrudes and solids (Figure S5); PCA of biocrudes (Figure S6); ATR-FTIR spectra of biocrudes (Figure S7); and photograph of the aqueous phases (Figure S8) (PDF)

## References

[1] United Nations Environment Programme . Global Waste Management Outlook 2024 - Beyond an Age of Waste: Turning Rubbish into a Resource; United Nations Environment Programme, 2024; 10.59117/20.500.11822/44939.

[2] Shahbeik H., Kazemi Shariat Panahi H., Dehhaghi M., Guillemin G. J., Fallahi A., Hosseinzadeh-Bandbafha H., Amiri H., Rehan M., Raikwar D., Latine H., Pandalone B., Khoshnevisan B., Sonne C., Vaccaro L., Nizami A. S., Gupta V. K., Lam S. S., Pan J., Luque R., Sels B., Peng W., Tabatabaei M., Aghbashlo M. (2024). Biomass to Biofuels Using Hydrothermal Liquefaction:
A Comprehensive Review. Renew. Sustain. Energy
Rev..

[3] Mathanker A., Das S., Pudasainee D., Khan M., Kumar A., Gupta R. (2021). A Review of
Hydrothermal Liquefaction of Biomass for Biofuels Production with
a Special Focus on the Effect of Process Parameters, Co-Solvents and
Extraction Solvents. Energies.

[4] Mishra R. K., Kumar V., Kumar P., Mohanty K. (2022). Hydrothermal Liquefaction
of Biomass for Bio-Crude Production: A Review on Feedstocks, Chemical
Compositions, Operating Parameters, Reaction Kinetics, Techno-Economic
Study, and Life Cycle Assessment. Fuel.

[5] Marzbali M. H., Kundu S., Halder P., Patel S., Hakeem I. G., Paz-Ferreiro J., Madapusi S., Surapaneni A., Shah K. (2021). Wet Organic Waste Treatment
via Hydrothermal Processing: A Critical
Review. Chemosphere.

[6] Selvam, A. ; Ilamathi, P. M. K. ; Udayakumar, M. ; Murugesan, K. ; Banu, J. R. ; Khanna, Y. ; Wong, J. Food Waste Properties. In Current Developments in Biotechnology and Bioengineering; Elsevier, 2021; pp 11–41. 10.1016/B978-0-12-819148-4.00002-6.

[7] Prestigiacomo, C. ; Scialdone, O. ; Galia, A. Hydrothermal Liquefaction of Wet Biomass in Batch Reactors: Critical Assessment of the Role of Operating Parameters as a Function of the Nature of the Feedstock. Journal of Supercritical Fluids.; Elsevier B.V., 2022; p 105689. 10.1016/j.supflu.2022.105689.

[8] Toor S. S., Rosendahl L., Rudolf A. (2011). Hydrothermal Liquefaction
of Biomass:
A Review of Subcritical Water Technologies. Energy.

[9] Tito E., Marcolongo C. A., Pipitone G., Monteverde A. H. A., Bensaid S., Pirone R. (2024). Understanding the Effect of Heating
Rate on Hydrothermal Liquefaction: A Comprehensive Investigation from
Model Compounds to a Real Food Waste. Bioresour.
Technol..

[10] Biller P., Ross A. B. (2011). Potential Yields and Properties of Oil from the Hydrothermal
Liquefaction of Microalgae with Different Biochemical Content. Bioresour. Technol..

[11] Déniel M., Haarlemmer G., Roubaud A., Weiss-Hortala E., Fages J. (2017). Hydrothermal Liquefaction
of Blackcurrant Pomace and Model Molecules:
Understanding of Reaction Mechanisms. Sustain.
Energy Fuels.

[12] Ahmad F., Doddapaneni T. R. K. C., Toor S. S., Kikas T. (2025). Reaction Mechanism
and Kinetics of Hydrothermal Liquefaction at Sub- and Supercritical
Conditions: A Review. Biomass (Switzerland).

[13] Leng L., Zhang W., Peng H., Li H., Jiang S., Huang H. (2020). Nitrogen in Bio-Oil Produced from
Hydrothermal Liquefaction of Biomass:
A Review. Chem. Eng. J..

[14] Duan P., Savage P. E. (2011). Hydrothermal Liquefaction
of a Microalga with Heterogeneous
Catalysts. Ind. Eng. Chem. Res..

[15] Cheng F., Tompsett G. A., Murphy C. M., Maag A. R., Carabillo N., Bailey M., Hemingway J. J., Romo C. I., Paulsen A. D., Yelvington P. E., Timko M. T. (2020). Synergistic Effects of Inexpensive
Mixed Metal Oxides for Catalytic Hydrothermal Liquefaction of Food
Wastes. ACS Sustain. Chem. Eng..

[16] Motavaf B., Capece S. H., Savage P. E. (2021). Screening Potential Catalysts for
the Hydrothermal Liquefaction of Food Waste. Energy Fuels.

[17] Nallasivam J., Francis Prashanth P., Harisankar S., Nori S., Suryanarayan S., Chakravarthy S. R., Vinu R. (2022). Valorization of Red Macroalgae Biomass
via Hydrothermal Liquefaction Using Homogeneous Catalysts. Bioresour. Technol..

[18] Liu Q., Zhang G., Liu M., Kong G., Xu R., Han L., Zhang X. (2022). Fast Hydrothermal
Liquefaction Coupled with Homogeneous
Catalysts to Valorize Livestock Manure for Enhanced Biocrude Oil and
Hydrochar Production. Renew. Energy.

[19] Koley S., Khadase M. S., Mathimani T., Raheman H., Mallick N. (2018). Catalytic
and Non-Catalytic Hydrothermal Processing of Scenedesmus Obliquus
Biomass for Bio-Crude Production – A Sustainable Energy Perspective. Energy Convers. Manag..

[20] Yang J., Nasirian N., Chen H., Niu H., He Q. (2022). Hydrothermal
Liquefaction of Sawdust in Seawater and Comparison between Sodium
Chloride and Sodium Carbonate. Fuel.

[21] Zhang L., Wang J., Ming H., Hu H., Dou X., Xiao Y., Cheng L., Hu Z. (2024). Investigation
of Cotton
Stalk-Derived Hydrothermal Bio-Oil: Effects of Mineral Acid/Base and
Oxide Additions. Energies.

[22] Zhang B., He Z., Chen H., Kandasamy S., Xu Z., Hu X., Guo H. (2018). Effect of
Acidic, Neutral and Alkaline Conditions on Product Distribution
and Biocrude Oil Chemistry from Hydrothermal Liquefaction of Microalgae. Bioresour. Technol..

[23] Xu J., Dong X., Wang Y. (2020). Hydrothermal
Liquefaction of Macroalgae
over Various Solids, Basic or Acidic Oxides and Metal Salt Catalyst:
Products Distribution and Characterization. Ind. Crops Prod..

[24] Ding X., Subramanya S. M., Fang T., Guo Y., Savage P. E. (2020). Effects
of Potassium Phosphates on Hydrothermal Liquefaction of Triglyceride,
Protein, and Polysaccharide. Energy Fuels.

[25] Chen F., Wang Y., Zheng L., Wu L., Ding X. (2023). Hydrothermal
Liquefaction of Lignocellulosic Biomass with Potassium Phosphate and
Iron and Their Binary Mixture: A Comprehensive Investigation on the
Yields and Compositions of Biocrude and Solid Residue. Bioresour. Technol..

[26] Bozym M., Florczak I., Zdanowska P., Wojdalski J., Klimkiewicz M. (2015). An Analysis of Metal Concentrations in Food Wastes
for Biogas Production. Renew. Energy.

[27] Michel J., Rivas-Arrieta M. J., Borén E., Simonin L., Kennedy M., Dupont C. (2025). Fate of Biomass
Inorganic Elements during Hydrothermal
Carbonization: An Experimental Study on Agro-Food Waste. Biomass Convers. Biorefinery.

[28] Tito E., Landi D., Demichelis F., Pipitone G., Bensaid S., Pirone R. (2025). Hydrothermal Liquefaction
of Digestate from the Organic
Fraction of Municipal Solid Waste: Optimization of Operating Parameters. Energy Convers. Manag..

[29] Channiwala S. A., Parikh P. P. (2002). A Unified Correlation
for Estimating HHV of Solid,
Liquid and Gaseous Fuels. Fuel.

[30] Ballabio D. (2015). A MATLAB Toolbox
for Principal Component Analysis and Unsupervised Exploration of Data
Structure. Chemom. Intell. Lab. Syst..

[31] García-Sancho C., Fúnez-Núñez I., Moreno-Tost R., Santamaría-González J., Pérez-Inestrosa E., Fierro J. L. G., Maireles-Torres P. (2017). Beneficial
Effects of Calcium Chloride
on Glucose Dehydration to 5-Hydroxymethylfurfural in the Presence
of Alumina as Catalyst. Appl. Catal. B Environ..

[32] Combs E., Cinlar B., Pagan-Torres Y., Dumesic J. A., Shanks B. H. (2013). Influence
of Alkali and Alkaline Earth Metal Salts on Glucose Conversion to
5-Hydroxymethylfurfural in an Aqueous System. Catal. Commun..

[33] He C., Wang K., Giannis A., Yang Y., Wang J. Y. (2015). Products
Evolution during Hydrothermal Conversion of Dewatered Sewage Sludge
in Sub- and near-Critical Water: Effects of Reaction Conditions and
Calcium Oxide Additive. Int. J. Hydrogen Energy.

[34] Tito E., Pipitone G., Monteverde Videla A.
H. A., Bensaid S., Pirone R. (2023). Exploring HTL Pathways in Carbohydrate–Protein
Mixture: A Study on Glucose–Glycine Interaction. Biomass Convers. Biorefinery.

[35] Ceragioli G., Schuck C. E., Zoppi G., Pipitone G., Anastasakis K., Bensaid S., Pirone R., Biller P. (2025). Development of an Integrated
Hydrothermal Liquefaction and Wet Oxidation Process: A Pathway for
an Autothermal Biorefinery. J. Clean. Prod..

[36] Gollakota A. R. K., Kishore N., Gu S. (2018). A Review on Hydrothermal Liquefaction
of Biomass. Renew. Sustain. Energy Rev..

[37] Madsen R. B., Biller P., Jensen M. M., Becker J., Iversen B. B., Glasius M. (2016). Predicting the Chemical
Composition of Aqueous Phase
from Hydrothermal Liquefaction of Model Compounds and Biomasses. Energy Fuels.

[38] Zhu Z., Toor S. S., Rosendahl L., Yu D., Chen G. (2015). Influence
of Alkali Catalyst on Product Yield and Properties via Hydrothermal
Liquefaction of Barley Straw. Energy.

[39] Harisankar S., Francis Prashanth P., Nallasivam J., Vishnu Mohan R., Vinu R. (2021). Effects of Aqueous
Phase Recirculation on Product Yields and Quality
from Hydrothermal Liquefaction of Rice Straw. Bioresour. Technol..

[40] Kumar M., Olajire Oyedun A., Kumar A. (2018). A Review on the Current Status of
Various Hydrothermal Technologies on Biomass Feedstock. Renew. Sustain. Energy Rev..

[41] Kang S., Zhang S., Wang Z., Li S., Zhao F., Yang J., Zhou L., Deng Y., Sun G., Yu H. (2023). Highly Efficient Catalytic Pyrolysis of Oil Shale by
CaCl2 in Subcritical
Water. Energy.

[42] Yoshida H., Terashima M., Takahashi Y. (1999). Production of Organic Acids and Amino
Acids from Fish Meat by Sub-Critical Water Hydrolysis. Biotechnol. Prog..

[43] Zhu Z., Liu Z., Zhang Y., Li B., Lu H., Duan N., Si B., Shen R., Lu J. (2016). Recovery of Reducing Sugars and Volatile
Fatty Acids from Cornstalk at Different Hydrothermal Treatment Severity. Bioresour. Technol..

[44] Fu X., Liao Y., Glein C. R., Jamison M., Hayes K., Zaporski J., Yang Z. (2020). Direct Synthesis
of Amides from Amines
and Carboxylic Acids under Hydrothermal Conditions. ACS Earth Sp. Chem..

[45] Fu X., Liao Y., Aspin A., Yang Z. (2020). Effect of Copper Salts
on Amide Hydrothermal Formation and Reactivity. ACS Earth Sp. Chem..

[46] Lin X., Ye W., Mao Y., Li Z., Lan Q., He Q., Kang K., Zhang L., Shui T., Wu Y., Zhong X., Yang J. (2024). Role of Sea
Salt in Modulating Biomass-to-Biocrude
Conversion via Hydrothermal Liquefaction. Desalination.

