# Supplementary material for: Effects of Inorganics during Hydrothermal Liquefaction of Waste: A Comprehensive Study
Source: Energy Fuels. 2026 Feb 9;40(7):3647–62. doi: 10.1021/acs.energyfuels.5c05808 (PMC12928205; doi:10.1021/acs.energyfuels.5c05808)
Supplement: Supplementary file 1 [file ef5c05808_si_001.pdf]

SUPPORTING INFORMATION

Effects of Inorganics during Hydrothermal Liquefaction of Waste: a Comprehensive Study

Edoardo Tito, Marco Vitale, Giuseppe Pipitone*, Samir Bensaid, Raffaele Pirone

Department of Applied Science and Technology, Politecnico di Torino, Corso Duca degli Abruzzi 24, 10129, Turin, Italy.

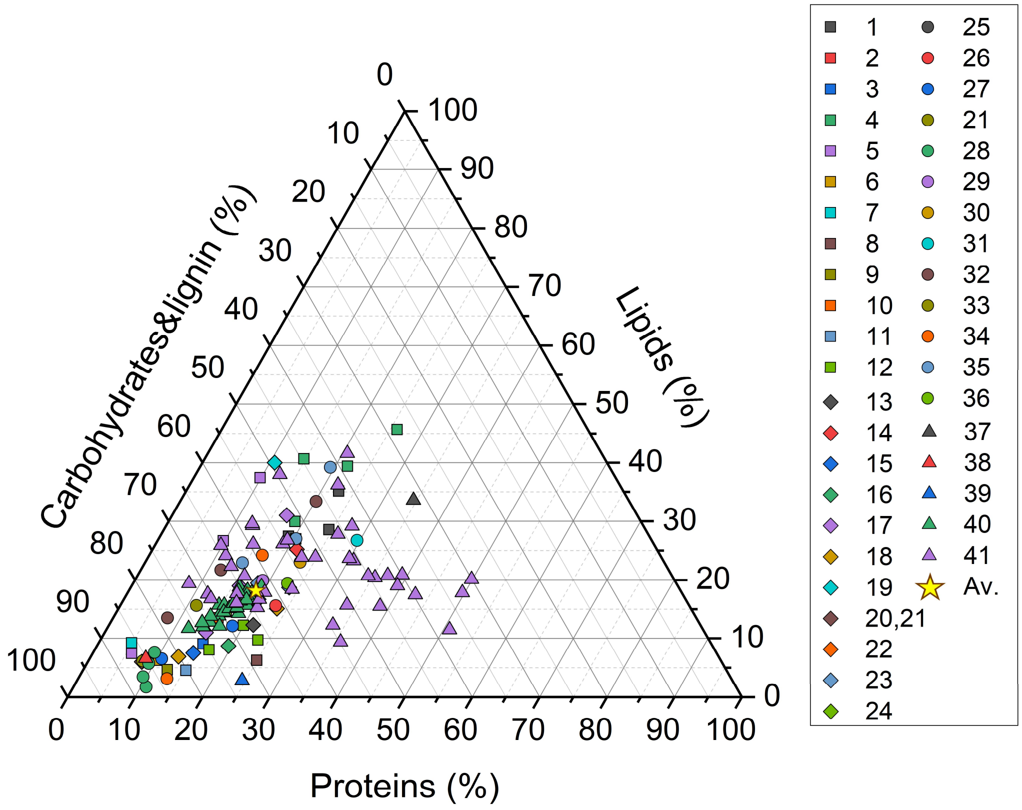

Figure S1. *Biochemical composition (proteins, lipids and carbohydrates & lignin) of food waste and the organic fraction of municipal solid waste, as reported in various studies, along with their average value (indicated by the star point). The studies are cited in the bibliography as follows: 1.[1] 2.[2] 3.[3] 4.[4] 5.[5] 6.[6] 7.[7] 8.[8] 9.[9] 10.[10] 11.[11] 12.[12] 13.[13] 14.[14] 15.[15] 16.[16] 17.[17] 18.[18] 19.[19] 20.[20] 21.[21] 22.[22] 23.[23] 24.[24] 25.[25] 26.[26] 27.[27] 28.[28] 29.[29] 30.[30] 31.[31] 32.[32] 33.[33] 34.[34] 35.[35] 36.[36] 37.[37] 38.[38] 39.[39] 40.[40] 41.[41]. For consistency across all studies, the amount of carbohydrates plus lignin (carbohydrates & lignin) was calculated by difference.*

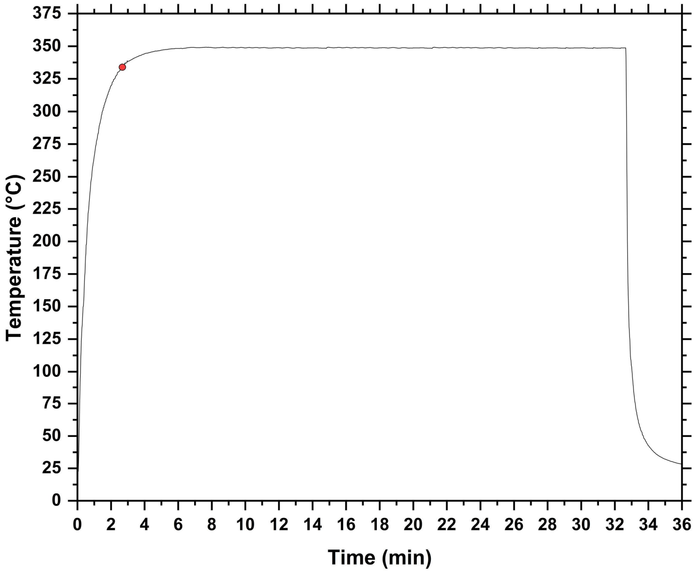

Figure S2. *Temperature profile inside the reactor during the reaction. The red dot represents the time at which the 30-minute waiting period began.*

Table S1. *$CaCO_3$ concentration and yield in final ash.*

Feed	$CaCO_3$ in final ash (g $CaCO_3$/g ash)	$CaCO_3$ yield (mol $CaCO_3$ final ash/mol Ca feed)
CaO	97 ± 1%	79 ± 11%
$CaCO_3$	95 ± 2%	67 ± 10%
$CaSO_4$	0 ± 0%	0 ± 0%
$CaCl_2$	54 ± 7%	11 ± 1%

Table S2. *Gas composition obtained from HTL test with CaO as inorganic.*

Compound	Content (vol%)	Content (wt%)
H_2	0.77%	0.04%
CO	2.47%	1.61%
CO_2	94.56%	96.90%
CH_4	1.00%	0.37%
C_2H_6	0.47%	0.33%
C_3H_8	0.73%	0.75%

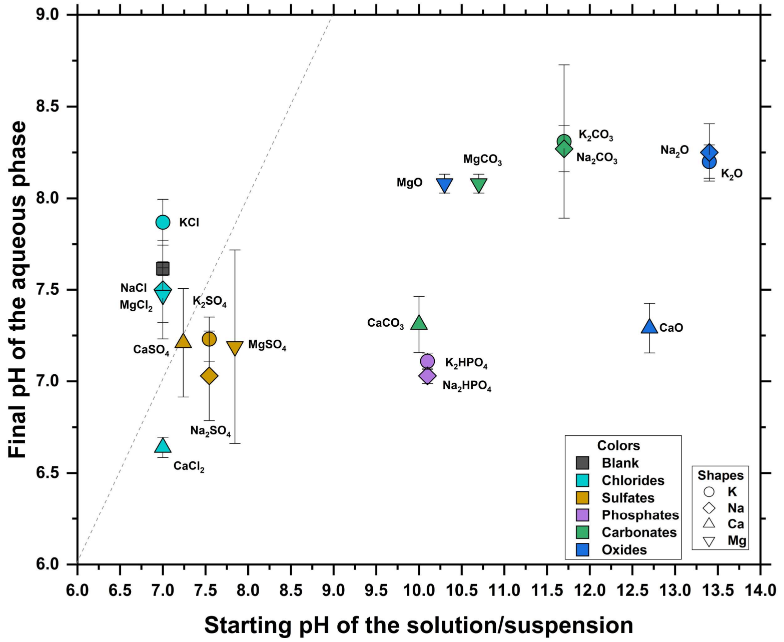

Figure S3. *Final pH of the aqueous phase compared to the starting theoretical pH of the solution/suspension as reported in Table 1. The shape of the symbols refers to the cation, while the color refers to the anion, as shown in the legends. Vertical error bars refer to the standard deviation of measurements performed at least in triplicate. The dashed line represents the graph diagonal.*

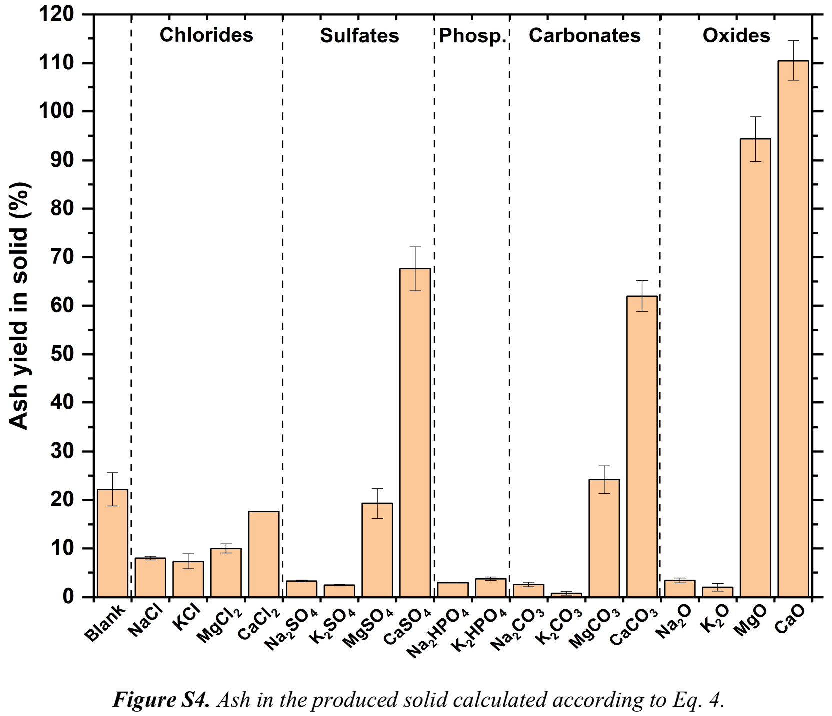

Figure S4. *Ash in the produced solid calculated according to Eq. 4.*

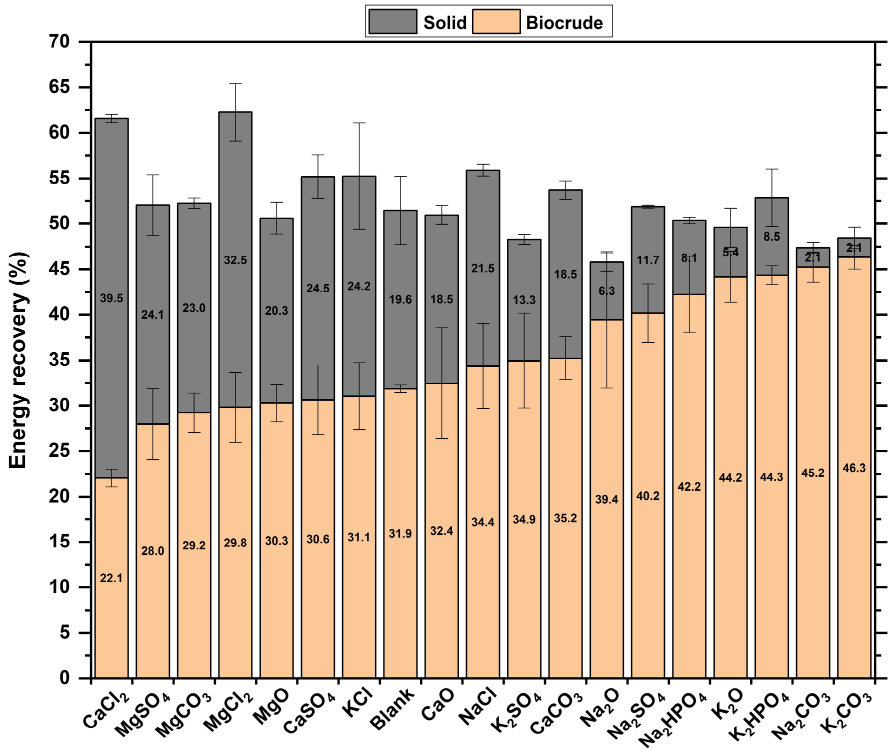

Figure S5. *Energy recoveries from Figure 6, rearranged in order of increasing biocrude energy recovery. Error bars refer to the standard deviation of experiments performed at least in triplicate.*

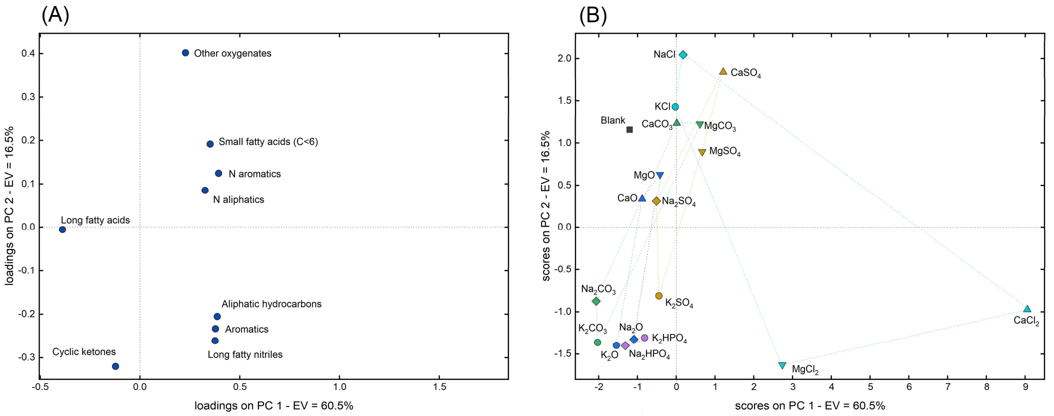

Figure S6. *Loadings (A) and scores (B) obtained from the principal component analysis (PCA) of the compound families of biocrudes shown in Figure7. EV denotes the explained variance of each principal component (PC) shown. The symbols used for the inorganic compounds in the scores plot are subdivided according to their cations and anions. Anion subdivision is based on color: oxides (blue), carbonates (green), hydrogen phosphates (purple), sulfates (mustard yellow), and chlorides (light blue). Cation subdivision is based on shape: K (circle), Na (diamond), Ca (upward-pointing triangle), and Mg (downward-pointing triangle).*

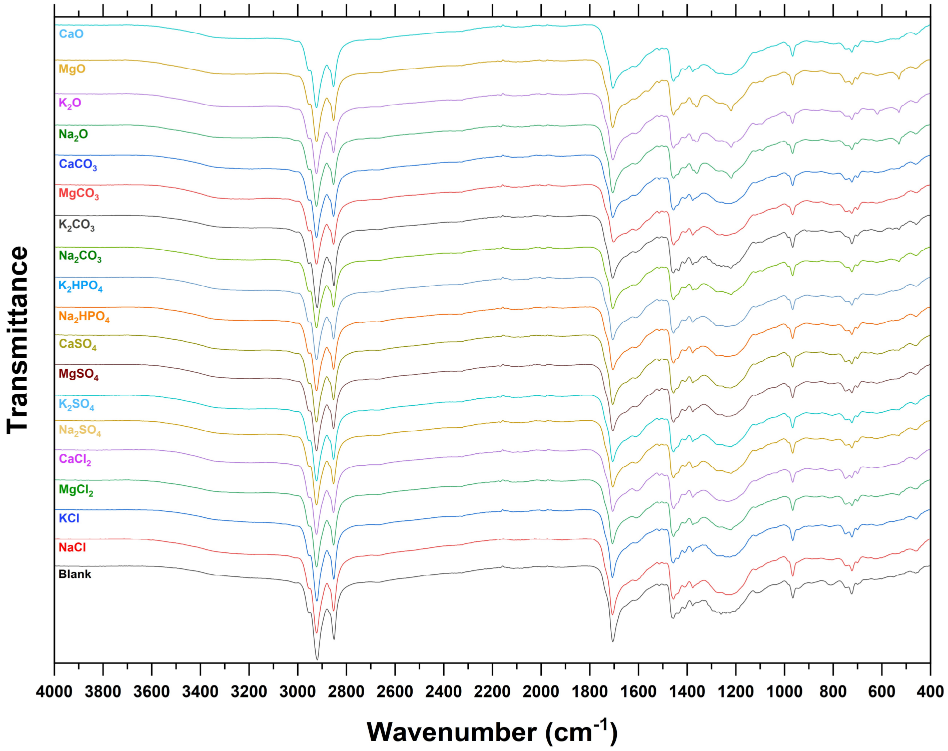

Figure S7. *ATR-FTIR spectra of the biocrudes obtained from all the tests performed.*

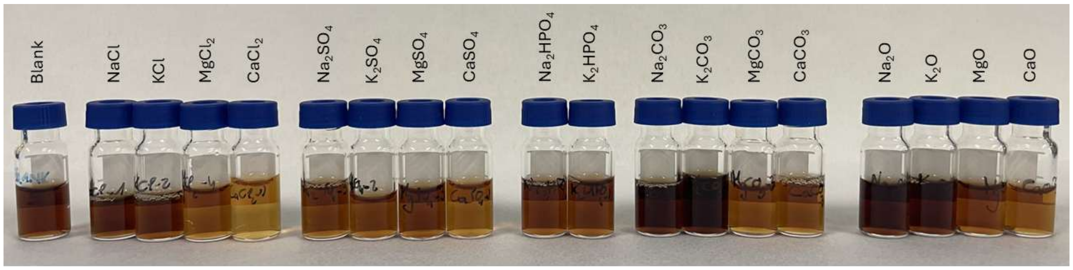

Figure S8. *Picture of the aqueous phases obtained from all the test performed.*

Bibliography

(1) Carmona-Cabello, M.; García, I. L.; Sáez-Bastante, J.; Pinzi, S.; Koutinas, A. A.; Dorado, M. P. Food Waste from Restaurant Sector – Characterization for Biorefinery Approach. *Bioresour. Technol.* **2020**, *301* (September 2019). https://doi.org/10.1016/j.biortech.2020.122779.

(2) Demichelis, F.; Pleissner, D.; Fiore, S.; Mariano, S.; Navarro Gutiérrez, I. M.; Schneider, R.; Venus, J. Investigation of Food Waste Valorization through Sequential Lactic Acid Fermentative Production and Anaerobic Digestion of Fermentation Residues. *Bioresour. Technol.* **2017**, *241*, 508–516. https://doi.org/10.1016/j.biortech.2017.05.174.

(3) Poe, N. E.; Yu, D.; Jin, Q.; Ponder, M. A.; Stewart, A. C.; Ogejo, J. A.; Wang, H.; Huang, H. Compositional Variability of Food Wastes and Its Effects on Acetone-

Butanol-Ethanol Fermentation. *Waste Manag.* **2020**, *107*, 150–158. https://doi.org/10.1016/j.wasman.2020.03.035.

(4) Lopez, V. M.; De la Cruz, F. B.; Barlaz, M. A. Chemical Composition and Methane Potential of Commercial Food Wastes. *Waste Manag.* **2016**, *56*, 477–490. https://doi.org/10.1016/j.wasman.2016.07.024.

(5) Pleissner, D.; Kwan, T. H.; Lin, C. S. K. Fungal Hydrolysis in Submerged Fermentation for Food Waste Treatment and Fermentation Feedstock Preparation. *Bioresour. Technol.* **2014**, *158* (2014), 48–54. https://doi.org/10.1016/j.biortech.2014.01.139.

(6) Ma, Y.; Cai, W.; Liu, Y. An Integrated Engineering System for Maximizing Bioenergy Production from Food Waste. *Appl. Energy* **2017**, *206* (August), 83–89. https://doi.org/10.1016/j.apenergy.2017.08.190.

(7) Ye, Z. L.; Zheng, Y.; Li, Y. H.; Cai, W. M. Use of Starter Culture of Lactobacillus Plantarum BP04 in the Preservation of Dining-Hall Food Waste. *World J. Microbiol. Biotechnol.* **2008**, *24* (10), 2249–2256. https://doi.org/10.1007/s11274-008-9737-z.

(8) Fisgativa, H.; Tremier, A.; Saoudi, M.; Le Roux, S.; Dabert, P. Biochemical and Microbial Changes Reveal How Aerobic Pre-Treatment Impacts Anaerobic Biodegradability of Food Waste. *Waste Manag.* **2018**, *80*, 119–129. https://doi.org/10.1016/j.wasman.2018.09.011.

(9) Liu, H.; Ma, J.; Wang, M.; Wang, W.; Deng, L.; Nie, K.; Yue, X.; Wang, F.; Tan, T. Food Waste Fermentation to Fumaric Acid by Rhizopus Arrhizus RH7-13. *Appl. Biochem. Biotechnol.* **2016**, *180* (8), 1524–1533. https://doi.org/10.1007/s12010-016-2184-7.

(10) Taheri, M. E.; Salimi, E.; Saragas, K.; Novakovic, J.; Barampouti, E. M.; Mai, S.; Malamis, D.; Moustakas, K.; Loizidou, M. Effect of Pretreatment Techniques on Enzymatic Hydrolysis of Food Waste. *Biomass Convers. Biorefinery* **2021**, *11* (2), 219–226. https://doi.org/10.1007/s13399-020-00729-7.

(11) Huang, H.; Singh, V.; Qureshi, N. Butanol Production from Food Waste: A Novel Process for Producing Sustainable Energy and Reducing Environmental Pollution. *Biotechnol. Biofuels* **2015**, *8* (1), 1–12. https://doi.org/10.1186/s13068-015-0332-x.

(12) Sayeki, M.; Toshinori, K.; Matsumoto, M.; Nishiyama, A.; Miyoshi, K.; Mochizuki, M.; Takasu, A.; Abe, A. Chemical Composition and Energy Value of Dried Meal from Food Waste as Feedstuff in Swine and Cattle. *Anim. Sci. J.* **2001**, *72* (7), J34–J40.

(13) Bake, G. G.; Endo, M.; Akimoto, A.; Takeuchi, T. Evaluation of Recycled Food Waste as a Partial Replacement of Fishmeal in Diets for the Initial Feeding of Nile Tilapia Oreochromis Niloticus. *Fish. Sci.* **2009**, *75* (5), 1275–1283. https://doi.org/10.1007/s12562-009-0133-x.

(14) Nasser, N.; Babikian, J.; Hatem, M. G.; Saoud, I. P.; Abiad, M. G. Evaluation of Post-Consumer Food Waste as Partial Replacement of Commercial Feed in

Marbled Rabbitfish, Siganus Rivulatus Aquaculture. *Int. J. Environ. Sci. Technol.* **2019**, *16* (8), 4059–4068. https://doi.org/10.1007/s13762-018-2051-x.

(15) Deka, R. S.; Kayastha, T. B.; Godara, R.; Goyal, B. M. Cost Economics of Raising Feeder Piglets on Kitchen Waste and Concentrate Ration. *Vet. Pract.* **2011**, *12* (1), 35–37.

(16) Hossein, S.; Dahlan, I. Growth Performance of Free-Range Village Chickens Fed Dehydrated Processed Food Waste. *J. Anim. Sci* **2015**, *18* (1), 77–86.

(17) Alibardi, L.; Cossu, R. Composition Variability of the Organic Fraction of Municipal Solid Waste and Effects on Hydrogen and Methane Production Potentials. *Waste Manag.* **2015**, *36*, 147–155. https://doi.org/10.1016/j.wasman.2014.11.019.

(18) Zhang, Y.; Banks, C. J.; Heaven, S. Anaerobic Digestion of Two Biodegradable Municipal Waste Streams. *J. Environ. Manage.* **2012**, *104*, 166–174. https://doi.org/10.1016/j.jenvman.2012.03.043.

(19) Moreno, A. D.; Magdalena, J. A.; Oliva, J. M.; Greses, S.; Coll Lozano, C.; Latorre-Sánchez, M.; Negro, M. J.; Susmozas, A.; Iglesias, R.; Llamas, M.; Tomás-Pejó, E.; González-Fernández, C. Sequential Bioethanol and Methane Production from Municipal Solid Waste: An Integrated Biorefinery Strategy towards Cost-Effectiveness. *Process Saf. Environ. Prot.* **2021**, *146*, 424–431. https://doi.org/10.1016/j.psep.2020.09.022.

(20) Barampouti, E. M.; Mai, S.; Malamis, D.; Moustakas, K.; Loizidou, M. Liquid Biofuels from the Organic Fraction of Municipal Solid Waste: A Review. *Renew. Sustain. Energy Rev.* **2019**, *110* (April), 298–314. https://doi.org/10.1016/j.rser.2019.04.005.

(21) Mahmoodi, P.; Karimi, K.; Taherzadeh, M. J. Hydrothermal Processing as Pretreatment for Efficient Production of Ethanol and Biogas from Municipal Solid Waste. *Bioresour. Technol.* **2018**, *261*, 166–175. https://doi.org/10.1016/j.biortech.2018.03.115.

(22) Mlaik, N.; Khoufi, S.; Hamza, M.; Masmoudi, M. A.; Sayadi, S. Enzymatic Pre-Hydrolysis of Organic Fraction of Municipal Solid Waste to Enhance Anaerobic Digestion. *Biomass and Bioenergy* **2019**, *127* (July). https://doi.org/10.1016/j.biombioe.2019.105286.

(23) Papa, G.; Scaglia, B.; D'Imporzano, G.; Savoldelli, S.; Jucker, C.; Colombini, S.; Toschi, I.; Adani, F. Valorizing the Organic Fraction of Municipal Solid Waste by Producing Black Soldier Fly Larvae and Biomethane in a Biorefinery Approach. *J. Clean. Prod.* **2022**, *379* (April). https://doi.org/10.1016/j.jclepro.2022.134422.

(24) Mahmoodi, P.; Karimi, K.; Taherzadeh, M. J. Efficient Conversion of Municipal Solid Waste to Biofuel by Simultaneous Dilute-Acid Hydrolysis of Starch and Pretreatment of Lignocelluloses. *Energy Convers. Manag.* **2018**, *166* (February), 569–578. https://doi.org/10.1016/j.enconman.2018.04.067.

(25) Shah, A. T.; Favaro, L.; Alibardi, L.; Cagnin, L.; Sandon, A.; Cossu, R.; Casella, S.; Basaglia, M. Bacillus Sp. Strains to Produce Bio-Hydrogen from the Organic

Fraction of Municipal Solid Waste. *Appl. Energy* **2016**, *176*, 116–124. https://doi.org/10.1016/j.apenergy.2016.05.054.

(26) Dong, L.; Zhenhong, Y.; Yongming, S. Semi-Dry Mesophilic Anaerobic Digestion of Water Sorted Organic Fraction of Municipal Solid Waste (WS-OFMSW). *Bioresour. Technol.* **2010**, *101* (8), 2722–2728. https://doi.org/10.1016/j.biortech.2009.12.007.

(27) Pecorini, I.; Baldi, F.; Carnevale, E. A.; Corti, A. Biochemical Methane Potential Tests of Different Autoclaved and Microwaved Lignocellulosic Organic Fractions of Municipal Solid Waste. *Waste Manag.* **2016**, *56*, 143–150. https://doi.org/10.1016/j.wasman.2016.07.006.

(28) Stylianou, E.; Pateraki, C.; Ladakis, D.; Cruz-Fernández, M.; Latorre-Sánchez, M.; Coll, C.; Koutinas, A. Evaluation of Organic Fractions of Municipal Solid Waste as Renewable Feedstock for Succinic Acid Production. *Biotechnol. Biofuels* **2020**, *13* (1), 1–16. https://doi.org/10.1186/s13068-020-01708-w.

(29) Browne, J. D.; Murphy, J. D. Assessment of the Resource Associated with Biomethane from Food Waste. *Appl. Energy* **2013**, *104*, 170–177. https://doi.org/10.1016/j.apenergy.2012.11.017.

(30) Helenas Perin, J. K.; Biesdorf Borth, P. L.; Torrecilhas, A. R.; Santana da Cunha, L.; Kuroda, E. K.; Fernandes, F. Optimization of Methane Production Parameters during Anaerobic Co-Digestion of Food Waste and Garden Waste. *J. Clean. Prod.* **2020**, *272*, 123130. https://doi.org/10.1016/j.jclepro.2020.123130.

(31) Browne, J. D.; Murphy, J. D. The Impact of Increasing Organic Loading in Two Phase Digestion of Food Waste. *Renew. Energy* **2014**, *71*, 69–76. https://doi.org/10.1016/j.renene.2014.05.026.

(32) Zhang, S.; Xiao, M.; Liang, C.; Chui, C.; Wang, N.; Shi, J.; Liu, L. Multivariate Insights into Enhanced Biogas Production in Thermophilic Dry Anaerobic Co-Digestion of Food Waste with Kitchen Waste or Garden Waste: Process Properties, Microbial Communities and Metagenomic Analyses. *Bioresour. Technol.* **2022**, *361* (May). https://doi.org/10.1016/j.biortech.2022.127684.

(33) Uçkun Kiran, E.; Trzcinski, A. P.; Liu, Y. Enhancing the Hydrolysis and Methane Production Potential of Mixed Food Waste by an Effective Enzymatic Pretreatment. *Bioresour. Technol.* **2015**, *183* (June), 47–52. https://doi.org/10.1016/j.biortech.2015.02.033.

(34) Sun, Y.; Wang, D.; Qiao, W.; Wang, W.; Zhu, T. Anaerobic Co-Digestion of Municipal Biomass Wastes and Waste Activated Sludge: Dynamic Model and Material Balances. *J. Environ. Sci. (China)* **2013**, *25* (10), 2112–2122. https://doi.org/10.1016/S1001-0742(12)60236-8.

(35) Peinemann, J. C.; Demichelis, F.; Fiore, S.; Pleissner, D. Techno-Economic Assessment of Non-Sterile Batch and Continuous Production of Lactic Acid from Food Waste. *Bioresour. Technol.* **2019**, *289* (April). https://doi.org/10.1016/j.biortech.2019.121631.

(36) Zhang, D.; Luo, W.; Liu, Y.; Yuan, J.; Li, G. Co-Biodrying of Sewage Sludge and

Organic Fraction of Municipal Solid Waste: A Thermogravimetric Assessment of the Blends. *Waste Manag.* **2019**, *95*, 652–660. https://doi.org/10.1016/j.wasman.2019.03.017.

(37) Cheng, J.; Ding, L.; Lin, R.; Liu, M.; Zhou, J.; Cen, K. Physicochemical Characterization of Typical Municipal Solid Wastes for Fermentative Hydrogen and Methane Co-Production. *Energy Convers. Manag.* **2016**, *117*, 297–304. https://doi.org/10.1016/j.enconman.2016.03.016.

(38) Farmanbordar, S.; Karimi, K.; Amiri, H. Municipal Solid Waste as a Suitable Substrate for Butanol Production as an Advanced Biofuel. *Energy Convers. Manag.* **2018**, *157* (November 2017), 396–408. https://doi.org/10.1016/j.enconman.2017.12.020.

(39) Biswas, J.; Chowdhury, R.; Bhattacharya, P. Kinetic Studies of Biogas Generation Using Municipal Waste as Feed Stock. *Enzyme Microb. Technol.* **2006**, *38* (3–4), 493–503. https://doi.org/10.1016/j.enzmictec.2005.07.004.

(40) Hansen, T. L.; Cour Jansen, J. la; Spliid, H.; Davidsson, Å.; Christensen, T. H. Composition of Source-Sorted Municipal Organic Waste Collected in Danish Cities. *Waste Manag.* **2007**, *27* (4), 510–518. https://doi.org/10.1016/j.wasman.2006.03.008.

(41) Esteves, S.; Devlin, D. *Chemical Characterisation of Food Wastes Collected from Welsh Local Authorities for Supporting Decisions Related to Anaerobic Digestion Process Design and Operation*; 2010. http://www.wrapcymru.org.uk/sites/files/wrap/Technical_report_food_waste_characterisation_Wales_2009x2.9086.pdf.
